# Efficacy and safety of Yangxin Dingji capsule as an adjunctive therapy for atrial fibrillation: a systematic review and meta-analysis of randomized controlled trials

**DOI:** 10.3389/fphar.2026.1952214

**Published:** 2026-10-14

**Authors:** Jingshuo Hao, Cong Ma, Jiaqi Ren, Ruoxi Ma, Yue Li, Jiaxiang Pan, Kaifeng Yu

**Affiliations:** 1 The First Clinical College, Liaoning University of Traditional Chinese Medicine, Shenyang, China; 2 School of Basic Medicine, Liaoning University of Traditional Chinese Medicine, Shenyang, China; 3 Department of Cardiology, Affiliated Hospital of Liaoning University of Traditional Chinese Medicine, Shenyang, China

**Keywords:** atrial fibrillation, meta-analysis, systematic review, traditional Chinese medicine, Yangxin DingJi capsule

## Abstract

**Background:**

Yangxin Dingji Capsule (YXDJ), a modern Chinese patent medicine derived from the classic Zhigancao Decoction, is commonly used as an adjuvant therapy for atrial fibrillation (AF). This study aimed to comprehensively evaluate the efficacy and safety of YXDJ as an adjuvant therapy in AF patients.

**Materials and Methods:**

We systematically searched PubMed, Embase, Web of Science, Cochrane Library, China Biology Medicine, Wanfang, VIP Chinese Journal Service Platform, and China National Knowledge Infrastructure from inception to 1 April 2026, for randomized controlled trials (RCTs) comparing YXDJ plus conventional therapy versus conventional therapy alone in patients with AF. Meta-analyses were performed using RevMan with fixed- or random-effects models based on heterogeneity. Risk of bias was assessed using the Cochrane Risk of Bias 2 (RoB 2) tool, and the overall quality of evidence for each outcome was evaluated using the GRADE framework. The protocol was registered in PROSPERO (CRD420261286691).

**Results:**

Ten RCTs involving 872 patients were included. All studies were Chinese-language publications with methodological limitations. According to the RoB 2 assessment, most studies were rated as having some concerns or high risk of bias, mainly due to inadequate allocation concealment, lack of blinding for subjective outcomes, and absence of publicly available trial registrations or protocols. In terms of clinical symptoms, YXDJ was associated with improvements in the overall clinical response rate [RR = 1.27, *P* < 0.00001, very low], reductions in EHRA symptom scores [MD = −0.47, *P* = 0.0009, very low], and reductions in postoperative AF recurrence at 6 months [RR = 0.43, *P* = 0.03, moderate]. For cardiac function, YXDJ was associated with increases in left atrial ejection fraction (LAEF) [MD = 2.11%, *P* = 0.003, low] and left ventricular ejection fraction (LVEF) [MD = 4.39%, *P* < 0.00001, moderate], whereas no significant changes were observed in left atrial diameter (LAD) [MD = −1.97 mm, *P* = 0.07, very low] and mitral E/A ratio [MD = 0.12, *P* = 0.06, very low]; subgroup analysis revealed that YXDJ reduced LAD in postoperative patients [MD = −2.70 mm, *P* = 0.02, low]. In terms of inflammatory biomarkers, YXDJ was associated with reductions in hs-CRP [MD = −1.21 mg/L, *P* < 0.00001, low], TNF-α [SMD = −0.44, *P* = 0.0009, low], and IL-6 [MD = −10.42 ng/L, *P* < 0.00001, low]; subgroup analysis further showed a greater reduction in hs-CRP in postoperative patients [MD = −1.42 mg/L, *P* < 0.00001, low]. For safety, no clear increase in the overall incidence of adverse events was observed [RR = 0.58, *P* = 0.07, low]. Exploratory analyses from single studies suggested possible long-term benefits, including a lower reported risk of ischemic stroke and sustained reductions in AF recurrence and symptom scores at 9 months post-ablation; however, these findings are preliminary and require confirmation.

**Conclusion:**

Current evidence, derived exclusively from methodologically limited, small, single-center Chinese studies, provides only preliminary signals that YXDJ may offer possible additional benefits as an adjunct to conventional therapy for symptom control and postoperative management in AF. Most efficacy outcomes were supported by low- or very-low-certainty evidence, with only LVEF and postoperative AF recurrence at 6 months rated as moderate certainty. These findings should be regarded as exploratory and hypothesis-generating. YXDJ should not replace guideline-directed anticoagulation, rate or rhythm control, or catheter ablation. Rigorously designed trials are needed to confirm its efficacy and safety.

**Systematic Review:**

Identifier CRD420261286691.

## 1 Introduction

Atrial fibrillation (AF) remains a prevalent cardiac arrhythmia and a major public health concern worldwide (Sun et al., 2025). In addition to impairing quality of life through symptoms such as palpitations, fatigue, and exercise intolerance, AF significantly increases the risk of stroke, heart failure, and all-cause mortality (Ruddox et al., 2017). Current management relies on antiarrhythmic drugs, anticoagulants, and catheter ablation. However, these approaches exhibit substantial interindividual variability in efficacy, are associated with adverse effects and long-term tolerability issues, and demonstrate relatively high post-procedural recurrence rates (Wolfes et al., 2025). Consequently, the development of effective and safe adjuvant or alternative therapeutic strategies is an urgent clinical priority for improving the management of AF.

Yangxin Dingji Capsule (YXDJ) is a modern Chinese patent medicine derived from the renowned classical formula *Zhigancao* Decoction, first documented in Zhang Zhongjing’s *Treatise on Febrile Diseases* (*Shanghan Lun*). In traditional Chinese medicine (TCM), this formula is specifically indicated for “palpitations with an intermittent and irregular pulse” (*Xin Dong Ji*, *Mai Jie Dai*), a clinical manifestation that closely corresponds to the modern definition of AF with ventricular arrhythmia. The formula comprises nine medicinal ingredients that collectively embody the TCM therapeutic principle of “supplementing *qi* and nourishing *yin*” to calm the heart. YXDJ (Approval No. Z19991082, produced by Shijiazhuang Pharmaceutical Group Hebei Yongfeng Pharmaceutical Co., Ltd., China) is a National Medical Products Administration (NMPA)-approved proprietary Chinese medicine, manufactured under Good Manufacturing Practice (GMP) conditions with quality controlled by national pharmaceutical standards. It is currently widely used in Chinese clinical practice as an adjuvant therapy for arrhythmias and has shown potential to improve symptoms and quality of life.

Previous clinical observations have suggested that YXDJ, as an adjuvant therapy to conventional antiarrhythmic treatment, may improve cardiac function and quality of life in AF patients. However, the existing individual randomized controlled trials (RCTs) are limited by small sample sizes and inconsistent outcome measures. Although a previous meta-analysis has synthesized the overall antiarrhythmic evidence for YXDJ (Wang XY. et al., 2021), it included heterogeneous arrhythmia populations rather than focusing specifically on AF, and its search was conducted several years ago. A substantial number of AF-specific RCTs have since been published, but these data have not been systematically integrated. Given the growing evidence base, an updated, AF-focused systematic review and meta-analysis is warranted to quantitatively synthesize the available data and assess the quality of current evidence. Therefore, we conducted this systematic review and meta-analysis to evaluate the efficacy and safety of YXDJ as an adjunctive therapy for AF, based on all available RCT evidence. We aimed to quantify the effects of YXDJ on clinical response, cardiac function, inflammatory markers, and arrhythmia recurrence, to critically appraise the methodological quality of the included studies, and to provide a potential evidence-based therapeutic option for integrating TCM into AF management.

## 2 Materials and methods

### 2.1 Protocol and registration

This systematic review was conducted and reported in accordance with the Preferred Reporting Items for Systematic Reviews and Meta-Analyses (PRISMA) 2020 statement (Page et al., 2021). The protocol was registered with the International Prospective Register of Systematic Reviews (PROSPERO) platform (https://www.crd.york.ac.uk/prospero/) under registration number CRD420261286691.

### 2.2 Information sources and search strategy

We systematically searched eight electronic databases from inception to 1 April 2026, to identify relevant studies on YXDJ for the treatment of AF. Databases included PubMed (https://pubmed.ncbi.nlm.nih.gov/), Embase (https://www.embase.com), Web of Science (WOS) (https://webofscience.clarivate.cn), Cochrane Library (https://www.cochranelibrary.com/), China Biology Medicine Database (CBM) (https://www.sinomed.ac.cn/), Wanfang Data (https://www.wanfangdata.com.cn/), VIP Chinese Journal Service Platform (VIP) (https://www.cqvip.com/), and China National Knowledge Infrastructure (CNKI) (https://www.cnki.net/). The search strategy combined Medical Subject Headings terms and free-text keywords, tailored to each database (Supplementary Material S1). No language restriction was applied to maximize retrieval sensitivity. We also screened the reference lists of relevant systematic reviews and meta-analyses. ClinicalTrials.gov and the WHO International Clinical Trials Registry Platform (ICTRP) were searched for ongoing or unpublished trials.

### 2.3 Inclusion and exclusion criteria

Studies were included based on the PICOS framework: (1) Population (P): adult patients diagnosed with AF, confirmed by electrocardiogram; (2) Intervention (I): treatment with YXDJ alone or YXDJ combined with conventional therapy; (3) Comparator (C): conventional therapy alone, placebo, or conventional therapy plus placebo; (4) Outcomes (O): studies reporting at least one of the predefined efficacy or safety outcome measures; (5) Study design (S): published RCTs. If the intervention group received combined therapy, the conventional therapy was required to be identical in dosage and route of administration to that received by the control group.

Studies were excluded if they (1) concurrently employed multiple other Traditional Chinese Medicine (TCM) therapies (including other Chinese patent medicines, other herbal decoctions, or non-pharmacological therapies such as acupuncture, moxibustion, massage, or acupoint injection) in the intervention group; (2) were non-randomized studies, reviews, case reports, or animal studies, conference abstracts, or dissertations/theses; or (3) had incomplete or unavailable data for outcome analysis.

### 2.4 Botanical nomenclature verification of botanical drug interventions

To comply with the recommended reporting standards for natural product-based pharmacological research (Heinrich et al., 2022), we performed a comprehensive taxonomic verification of all constituent medicinal materials of YXDJ as reported in the original RCTs. For each botanical ingredient, the current valid scientific name, author citation, and corresponding family were cross-checked using the Medicinal Plant Names Services (MPNS) hosted by the Royal Botanic Gardens, Kew, and the Plants of the World Online (POWO) database. For the single animal-derived component, namely, Donkey-hide Gelatin (Chinese name: E Jiao), which is not covered by botanical repositories, its zoological classification was verified against the Integrated Taxonomic Information System (ITIS) and relevant mammalian taxonomy references. All pharmaceutical (drug) names and designated medicinal parts were subsequently standardized according to the Pharmacopoeia of the People’s Republic of China (2025 Edition). The complete validated nomenclatural details for each ingredient are presented in Supplementary Material S2.1.

Unlike individually prepared aqueous decoctions, YXDJ is a nationally approved proprietary Chinese medicine (Approval No. Z19991082) manufactured by Shijiazhuang Pharmaceutical Group Hebei Yongfeng Pharmaceutical Co., Ltd., China, under Good Manufacturing Practice conditions. Its chemical composition is defined by the national drug standard (WS3-229(Z-229)-2002(Z)), which specifies HPLC determination of glycyrrhizic acid (not less than 0.50 mg per capsule) and TLC identification of ginsenoside Rg1 and cinnamaldehyde. All included RCTs reported the same manufacturer and approval number of YXDJ; however, specifications were not consistently reported, and most studies did not report the specification at all. In addition, none of the included RCTs reported batch numbers, detailed quality-control data, chemical fingerprints, marker compound content, or purity data of the YXDJ used. Because YXDJ is derived from botanical materials, its chemical composition may vary with the source and quality of raw herbs, harvest time, processing methods, and manufacturing conditions. These factors could not be assessed in the present review. Therefore, although the use of a standardized industrial product may improve consistency compared with individually prepared decoctions, batch-to-batch reproducibility across the included trials cannot be verified. The major active components of YXDJ and their chemical structures are summarized in Supplementary Material S2.2.

### 2.5 Outcome indicators

#### 2.5.1 Primary outcomes

Overall clinical response rate: The proportion of patients achieving “markedly effective” or “effective” status based on study-specific criteria. Although the definitions varied across trials, all were based on improvement in AF-related clinical symptoms, with some trials additionally incorporating objective cardiac parameters. We therefore pooled these data in the primary analysis and performed an exploratory subgroup analysis by definition type (symptom-based definitions vs. definitions incorporating objective cardiac assessments).

#### 2.5.2 Secondary outcomes

(1) Symptom burden: European Heart Rhythm Association (EHRA) symptom score; (2) Rhythm control: postoperative recurrence of AF at 6 months, defined as at least one documented episode of AF, atrial flutter, or atrial tachycardia lasting ≥30 s confirmed by 24-h Holter monitoring during the 6-month follow-up after ablation; (3) Cardiac structure and function (assessed by echocardiography): left atrial ejection fraction (LAEF), left ventricular ejection fraction (LVEF), left atrial diameter (LAD), and the mitral E/A ratio; (4) Inflammatory biomarkers: high-sensitivity C-reactive protein (hs-CRP), tumor necrosis factor-alpha (TNF-α), and interleukin-6 (IL-6); and (5) Safety: incidence of adverse events.

### 2.6 Data extraction

Two researchers trained in systematic review methodology independently performed literature screening and data extraction using standardized forms, and they were blinded to each other’s decisions. A third author subsequently verified the extracted data and resolved any discrepancies. Any disagreements were resolved through discussion or consultation with a third reviewer. An agreement statistic was not calculated. Extracted information included (1) study characteristics (first author, publication year, sample size, and study location); (2) participant information (age, sex, and type of AF); (3) intervention details (dosage, frequency, and duration of YXDJ, as well as the specific conventional therapy in the control group); (4) methodological features (randomization method, blinding procedures, and dropout and loss to follow-up rates); and (5) outcome data (all predefined primary and secondary outcomes).

### 2.7 Risk of bias assessment

Two researchers independently assessed the risk of bias for each main outcome of each included RCT using the Cochrane Risk of Bias 2 (RoB 2) tool. The RoB 2 covers five domains: (1) bias arising from the randomization process; (2) bias due to deviations from intended interventions; (3) bias due to missing outcome data; (4) bias in measurement of the outcome; and (5) bias in selection of the reported result. For each outcome, each domain and the overall risk of bias were judged as “low risk,” “some concerns,” or “high risk.” Cross-verification was performed, and disagreements were resolved through discussion or consultation with a senior researcher.

### 2.8 Data analysis

Descriptive summaries were provided for individual study outcomes. Outcomes were considered comparable if they used the same definition and measurement unit; for continuous biomarkers, assay methods were additionally taken into account when deciding between the mean difference (MD) and standardized mean difference (SMD). Meta-analyses of comparable outcomes were performed using RevMan 5.4.1. Continuous outcomes were analyzed using the MD or SMD, and dichotomous outcomes using risk ratios (RR), each with 95% confidence intervals (CI). Heterogeneity was assessed using the I^2^ statistic. A fixed-effects model was applied when I^2^ < 50% and the heterogeneity *P* > 0.10; otherwise, a random-effects model was used. When substantial heterogeneity (I^2^ ≥ 50%) was detected, sensitivity analyses were planned by sequentially excluding individual studies to explore potential sources. Exploratory subgroup analyses were pre-specified in consideration of the expected clinical heterogeneity across studies, including differences in surgical status (postoperative patients vs. non-surgical patients), AF subtype, background therapy, YXDJ dosage (<6.0 g/d vs. ≥ 6.0 g/d), and treatment duration (≤3 months vs. > 3 months). Where the number of studies allowed, subgroup analyses were performed; otherwise, heterogeneity was described narratively. Publication bias was planned to be assessed using funnel plots and Egger’s test for outcomes with ten or more studies (*n* ≥ 10).

### 2.9 GRADE assessment

The overall quality of evidence for each outcome was assessed using the Grading of Recommendations Assessment, Development and Evaluation (GRADE) framework (Guy et al., 2008). Evidence was rated as high, moderate, low, or very low based on five domains: risk of bias, inconsistency, indirectness, imprecision, and publication bias. Randomized controlled trials started as high-quality evidence and were downgraded based on the presence and severity of each limitation. Outcomes with substantial effect sizes (RR < 0.5 or >2.0) were considered for upgrading. Disagreements between reviewers were resolved through discussion.

## 3 Results

### 3.1 Search results and basic characteristics

A total of 109 articles were retrieved. After removing duplicates, 38 articles remained. Following the review of titles and abstracts, 20 articles were retained. After full-text assessment, 10 articles met the eligibility criteria and were included in the final analysis (Jiang and Lu, 2025; Liu et al., 2020; Xiao and Sun, 2020; Xue et al., 2020; Ying et al., 2021; Zhang et al., 2020; Zhang et al., 2019; Zhu and Zhang, 2023; Zhu and Zhang, 2024; Zhao, 2026) (Figure 1A). Among the 10 excluded articles, 1 was not an RCT, 6 used self-formulated preparations also named “Yangxin Dingji” (e.g., Yangxin Dingji Fang, Yangxin Dingji Tang, or Yangxin Dingji Chongji) but not the approved YXDJ capsule (Approval No. Z19991082), and 3 enrolled populations not limited to AF. All included RCTs were published in Chinese. We also screened the reference lists of relevant systematic reviews and meta-analyses, but no additional eligible studies were identified. No ongoing eligible RCTs were identified in trial registries as of 1 April 2026.

**FIGURE 1:**
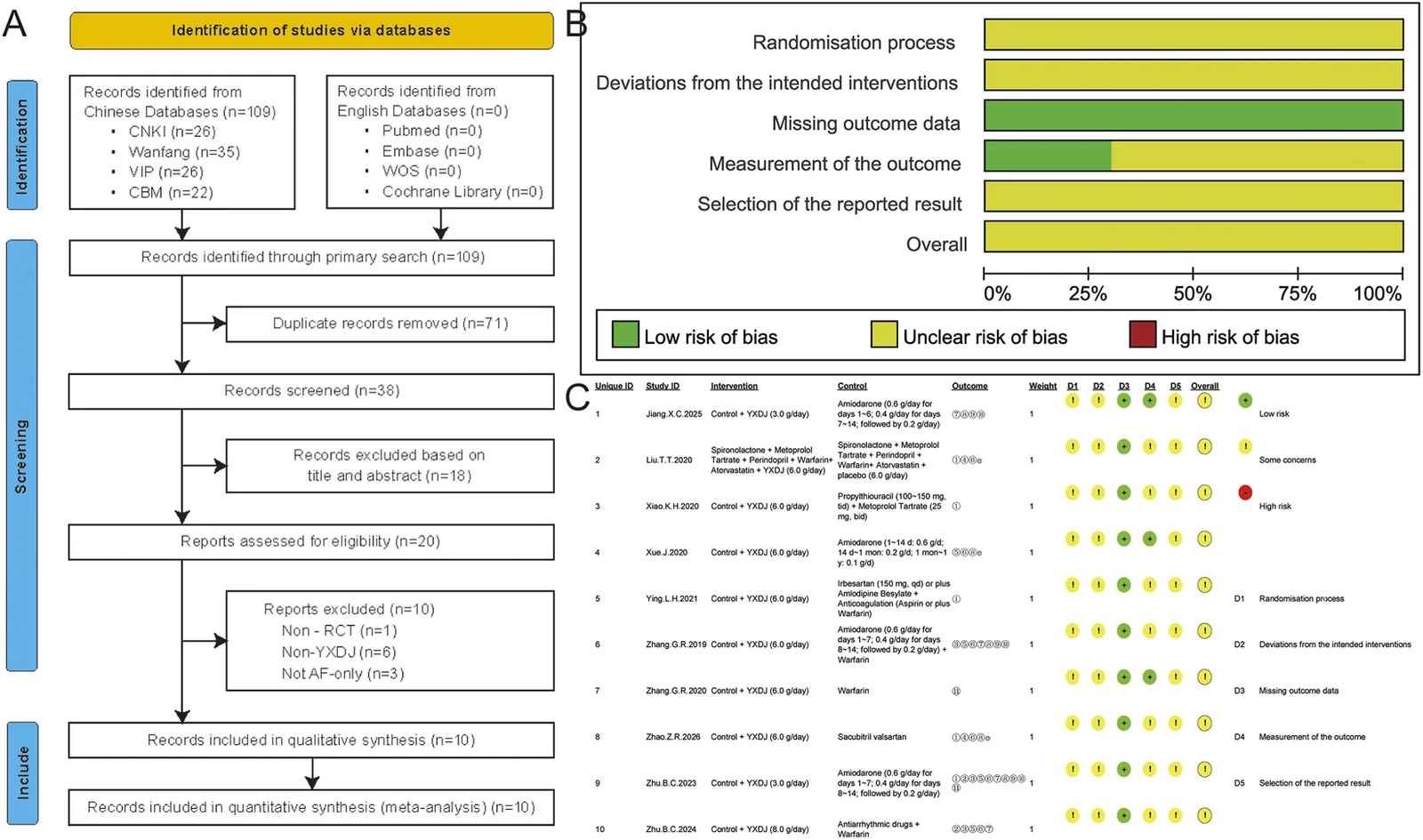
Baseline Characteristics and Risk of Bias Assessment of the Meta-Analysis. **(A)** Flowchart of the meta-analysis; **(B,C)** Risk of bias assessment for the included RCTs.

A total of 872 patients were included, 434 in the intervention group and 438 in the control group. All 10 trials were single-center studies conducted at hospitals in China, and all enrolled patients were of Chinese ethnicity. Therefore, the study populations were predominantly of East Asian descent, and none of the included trials reported detailed racial or ethnic composition. The duration of YXDJ treatment varied from 1 to 12 months, with 3 months or 12 weeks (*n* = 6) being the most common. The recommended daily dosage of YXDJ is 6–8 g according to the product label. However, the actual dosages used in the included trials varied: eight trials adhered to the recommended dosage (seven used 6.0 g/day (Liu et al., 2020; Xiao and Sun, 2020; Xue et al., 2020; Ying et al., 2021; Zhang et al., 2020; Zhang et al., 2019; Zhao, 2026) and one used 8.0 g/day (Zhu and Zhang, 2024)), while the remaining two trials used a lower dosage of 3.0 g/day (Jiang and Lu, 2025; Zhu and Zhang, 2023). The intervention group received YXDJ in addition to conventional treatment in all included studies.

Regarding post-treatment follow-up beyond the treatment period, four studies reported extended observation after the completion of YXDJ administration: Zhang GR (2020) reported a mean follow-up of 10.7 ± 2.4 months for ischemic stroke events after a 1-month treatment course (Zhang et al., 2020); Zhu BC (2023) extended follow-up to 6 months post-ablation after a 3-month treatment (Zhu and Zhang, 2023); Zhang GR (2019) (Zhang et al., 2019) and Zhu BC (2024) (Zhu and Zhang, 2024) both extended follow-up to 9 months after a 3-month treatment for recurrence rates and symptom scores. By contrast, Xue J (2020) (Xue et al., 2020) had a 12-month treatment period with outcomes assessed at the end of the 12-month treatment course, rather than a post-treatment observational phase. The remaining studies assessed outcomes at the end of the treatment course without additional post-treatment observation. The basic characteristics of all RCTs included in the analysis are presented in Table 1.

**TABLE 1:** Basic characteristics of included studies.

Author (Year)	Sample size (Male/Female)	Age (I/C)	Types of AF	Measures		Duration/Follow-up	Outcomes
				Intervention	Control		
(Jiang and Lu, 2025)	I: 37 (20/17) C: 37 (21/16)	I: 60.24 ± 3.38 C: 60.32 ± 3.45	Post-radiofrequency ablation paroxysmal AF	Control + YXDJ (3.0 g/day)	Amiodarone (0.6 g/day for days 1–6; 0.4 g/day for days 7–14; followed by 0.2 g/day)	3 months/-	④⑧⑨⑩⑪
(Liu et al., 2020)	I: 52 (32/20) C: 52 (33/19)	I: 65.1 ± 4.5 C: 64.9 ± 5.3	Chronic heart failure and AF with prolonged R-R interval	Spironolactone + Metoprolol Tartrate + Perindopril + Warfarin + Atorvastatin + YXDJ (6.0 g/day)	Spironolactone + Metoprolol Tartrate + Perindopril + Warfarin + Atorvastatin + placebo (6.0 g/day)	12 weeks/-	①④⑥⑪
(Xiao and Sun, 2020)	I: 43 (18/25) C: 42 (19/23)	I: 41.02 ± 5.07 C: 40.28 ± 5.13	Hyperthyroidism-induced AF	Control + YXDJ (6.0 g/day)	Propylthiouracil (100–150 mg, tid) + Metoprolol Tartrate (25 mg, bid)	8 weeks/-	①
(Xue et al., 2020)	I: 33 (−) C: 34 (−)	I: 69.32 ± 5.44 C: 68.37 ± 5.36	Paroxysmal AF	Control + YXDJ (6.0 g/day)	Amiodarone (1–14 d: 0.6 g/d; 14 d–1 mon: 0.2 g/d; 1 mon∼1 y: 0.1 g/d)	12 months/-	⑤⑥⑧⑪
(Ying et al., 2021)	I: 63 (36/27)/C: 63 (34/29)	I: 57.02 ± 5.49 C: 56.14 ± 5.06	Hypertension and Paroxysmal AF	Control + YXDJ (6.0 g/day)	Irbesartan (150 mg, qd) or plus Amlodipine Besylate + Anticoagulation (Aspirin or plus Warfarin)	2 months/-	①
(Zhang et al., 2020)	I: 50 (27/23) C: 59 (34/25)	I: 73.3 ± 12.1 C: 73.9 ± 13.2	Non-valvular paroxysmal AF	Control + YXDJ (6.0 g/day)	Warfarin	1 month/(10.7 ± 2.4) months	⑪
(Zhang et al., 2019)	I: 34 (19/15) C: 34 (20/14)	I: 65.2 ± 6.4 C: 64.1 ± 6.5	Post-radiofrequency ablation persistent and paroxysmal AF	Control + YXDJ (6.0 g/day)	Amiodarone (0.6 g/day for days 1–7; 0.4 g/day for days 8–14; followed by 0.2 g/day) + Warfarin	3 months/9 months	②③⑤⑥⑦⑧⑨⑩
(Zhu and Zhang, 2023)	I: 44 (23/21) C: 44 (24/20)	I: 66.55 ± 4.62 C: 66.53 ± 4.52	Post-radiofrequency ablation paroxysmal AF	Control + YXDJ (3.0 g/day)	Amiodarone (0.6 g/day for days 1–7; 0.4 g/day for days 8–14; followed by 0.2 g/day)	3 months/6 months	①②③⑤⑥⑦⑧⑨⑩⑪
(Zhu and Zhang, 2024)	I: 35 (19/16) C: 30 (15/15)	I: 66.93 ± 7.11 C: 67.27 ± 5.37	Post-single ring pulmonary vein isolation paroxysmal AF	Control + YXDJ (8.0 g/day)	Antiarrhythmic drugs + Warfarin	3 months/9 months	②③⑤⑥⑦
(Zhao, 2026)	I: 43 (29/14) C: 43 (27/16)	I: 73.10 ± 5.80 C: 72.51 ± 6.24	Paroxysmal AF (surgical status not clearly defined)	Control + YXDJ (6.0 g/day)	Sacubitril valsartan	12 weeks/-	①④⑥⑧⑪

Follow-up duration is calculated from baseline to the final outcome assessment. ①Overall Clinical Response Rate; ②EHRA, symptom score; ③Postoperative Recurrence of AF, at 6 Months; ④LVEF; ⑤LAEF; ⑥LAD; ⑦Mitral E/A ratio; ⑧hs-CRP; ⑨TNF-α; ⑩IL-6; ⑪Adverse Reactions.

### 3.2 Baseline assessment

The baseline characteristics of the included studies are summarized in Table 1. The mean age ranged from 41.02 to 73.3 years in the intervention group and from 40.28 to 73.9 years in the control group. Male participants accounted for 51.1%–61.5% of the intervention groups and 50.0%–61.5% of the control groups across studies. Regarding AF type, four trials enrolled post-ablation patients (Jiang and Lu, 2025; Zhang et al., 2019; Zhu and Zhang, 2023; Zhu and Zhang, 2024), and six enrolled non-surgical AF populations, including patients with heart failure, hyperthyroidism, hypertension, and non-valvular AF. Because randomization is intended to balance baseline characteristics between groups, statistical testing of baseline variables was not performed.

### 3.3 Risk of bias evaluation

The risk of bias assessment using RoB 2 is summarized in Figures 1B,C. The risk of bias was assessed using the RoB 2 tool for each main outcome. (1) Randomization process: Nine RCTs used a random number table, and one did not describe the randomization method (Zhang et al., 2020); none of the studies reported allocation concealment, but baseline characteristics were balanced. Therefore, all were rated as “Some concerns.” (2) Deviations from intended interventions: Liu TT (2020) (Liu et al., 2020) used a placebo control with single blinding of participants but did not report whether personnel were blinded; the remaining nine RCTs were not blinded, and the different drug formulations made it likely that participants were aware of the intervention. Therefore, all were rated as “Some concerns.” (3) Missing outcome data: All studies completed the trial without dropout or loss to follow-up and reported outcome data for all patients, so this domain was rated as “Low.” (4) Outcome measurement: Objective outcomes (such as LVEF, LAEF, LAD, mitral E/A ratio, hs-CRP, TNF-α, IL-6, and postoperative recurrence of AF at 6 months) were measured by appropriate methods, and knowledge of the intervention was unlikely to influence measurement; therefore, these were rated as “Low.” Subjective outcomes (such as overall clinical response rate (Liu et al., 2020; Xiao and Sun, 2020; Ying et al., 2021; Zhu and Zhang, 2023; Zhao, 2026) and EHRA symptom score (Zhang et al., 2019; Zhu and Zhang, 2023; Zhu and Zhang, 2024)) were rated as “Some concerns” because outcome assessors were not blinded and measurement could have been influenced by knowledge of the intervention, although this was not considered highly likely. (5) Selection of the reported result: None of the studies provided a publicly available trial registration or prospective protocol, so it was impossible to confirm whether all prespecified outcomes had been fully reported; therefore, all were rated as “Some concerns.” In summary, for the main outcomes, the overall risk of bias was rated as “Some concerns” for all included RCTs.

### 3.4 Efficacy evaluation

#### 3.4.1 Symptom burden

##### 3.4.1.1 Overall clinical response rate

Five studies reported the overall clinical response rate (Liu et al., 2020; Xiao and Sun, 2020; Ying et al., 2021; Zhu and Zhang, 2023; Zhao, 2026). Although the specific criteria varied across trials, all definitions were based on improvement in clinical symptoms, with or without additional objective cardiac assessment. Liu TT (2020) (Liu et al., 2020), Xiao KH (2020) (Xiao and Sun, 2020), and Ying LH (2021) (Ying et al., 2021) defined response mainly according to changes in TCM symptom scores. Liu TT (2020) used a ≥90% reduction for “markedly effective” and >50% but <90% for “effective,” whereas Xiao KH (2020) (Xiao and Sun, 2020) and Ying LH (2021) (Ying et al., 2021) both used ≥70% for “markedly effective” and ≥30% but <70% for “effective.” Zhao ZR (2026) (Zhao, 2026) used a composite definition that included AF burden, sinus rhythm maintenance, left atrial diameter, TCM symptom score, and EHRA class. Zhu BC (2023) (Zhu and Zhang, 2023) defined “markedly effective” as complete resolution of symptoms with restoration of regular and ordered electrical activity on ECG, and “effective” as symptomatic improvement with improvement in ECG electrical activity. Despite these differences in thresholds and the inclusion of objective parameters in some trials, all definitions reflected improvement in AF-related clinical symptoms. We therefore pooled these data in the primary analysis.

In the primary meta-analysis, no significant heterogeneity was observed among the studies (I^2^ = 0%, *P* = 0.82). A fixed-effect meta-analysis revealed that YXDJ was associated with a statistically significant improvement in the overall clinical response rate compared with the control group [RR = 1.27, 95% CI (1.16, 1.40), *P* < 0.00001] (Figure 2A). A sensitivity analysis using a random-effects model yielded a consistent estimate [RR = 1.26, 95% CI (1.15, 1.39), *P* < 0.00001] (Supplementary Figure S1.1). This estimate represents a mixed population of one postoperative and four non-surgical studies; the postoperative subgroup could not be pooled separately.

**FIGURE 2:**
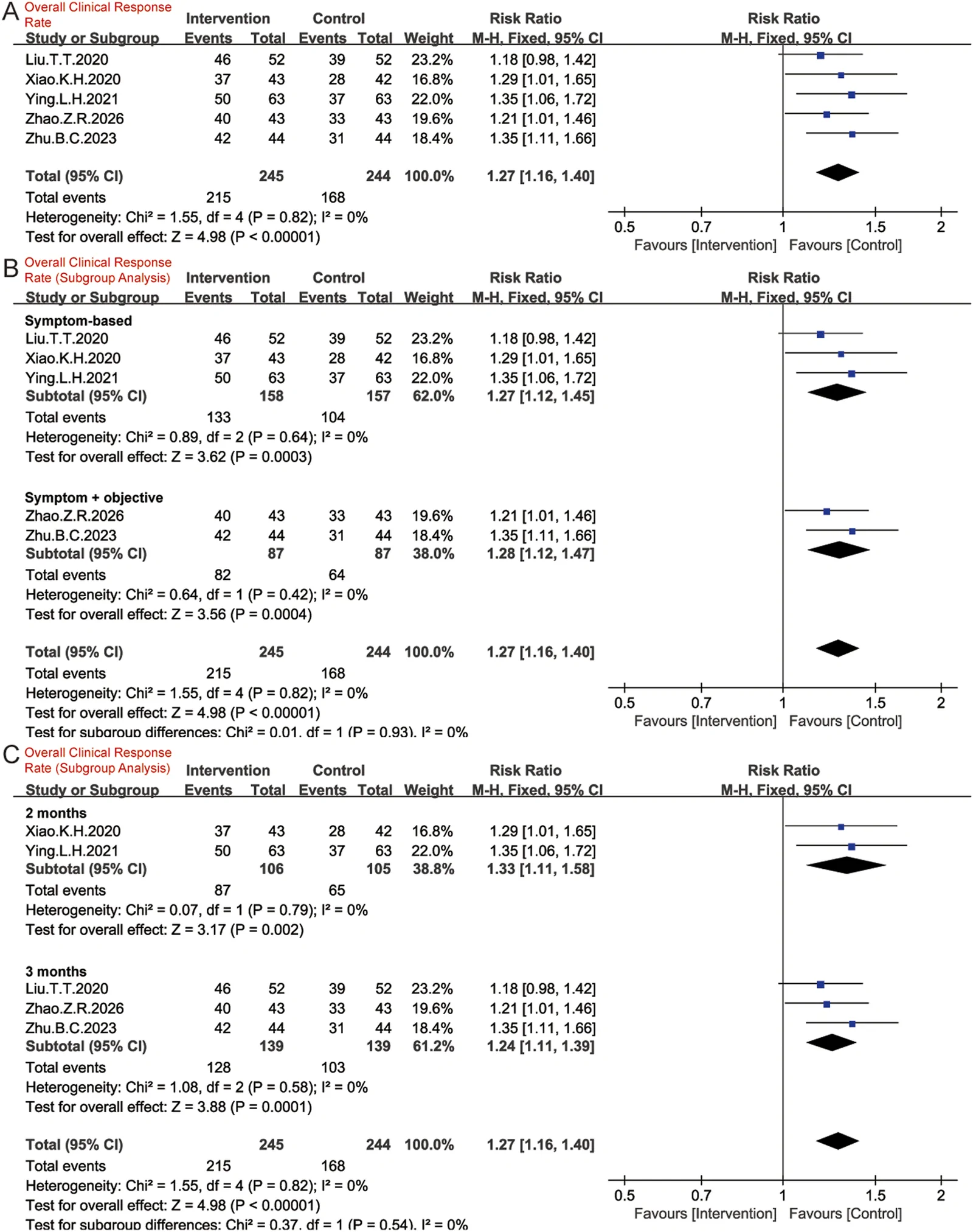
Forest Plots for the Effects of YXDJ on Overall Clinical Response Rate. **(A)** Pooled forest plot for overall clinical response rate. **(B)** Subgroup analysis of overall clinical response rate by definition type (symptom-based vs. symptom plus objective assessment). **(C)** Subgroup analysis of overall clinical response rate by treatment duration (2-month vs. 3-month).

To examine whether the definition of response influenced the pooled estimate, we performed an exploratory subgroup analysis by definition type. When the analysis was restricted to the three studies that defined response primarily by symptom scores (Liu et al., 2020; Xiao and Sun, 2020; Ying et al., 2021), the pooled RR was [I^2^ = 0%, fixed-effects model: RR = 1.27, 95% CI (1.12, 1.45), *P* = 0.0003]. When the analysis was restricted to the two studies that incorporated objective cardiac assessments (Zhu and Zhang, 2023; Zhao, 2026), the pooled RR was [I^2^ = 0%, fixed-effects model: RR = 1.28, 95% CI (1.12, 1.47), *P* = 0.0004] (Figure 2B). Both subgroups showed consistent beneficial effects. Because each subgroup contained only two to three studies, this subgroup analysis should be considered exploratory.

We also examined other potential sources of clinical heterogeneity, including surgical status, AF subtype, background therapy, YXDJ dosage, and treatment duration. Only one of the five studies was conducted in postoperative patients and used a low dose of 3.0 g/day (Zhu and Zhang, 2023), whereas the remaining four were non-surgical and used 6.0 g/day. Because surgical status and dosage differed only in this single study, a sensitivity analysis excluding Zhu BC 2023 (Zhu and Zhang, 2023) was performed. The remaining four studies were all conducted in non-surgical patients, so this analysis also represents the non-surgical subgroup. The pooled estimate for the remaining four studies remained consistent [I^2^ = 0%, fixed-effects model: RR = 1.26, 95% CI (1.13, 1.40), *P* < 0.0001] (Supplementary Figure S1.2), indicating that the overall result was not driven by surgical status or dosage. Two studies used a treatment duration of approximately 2 months (Xiao and Sun, 2020; Ying et al., 2021), and three used approximately 3 months (Liu et al., 2020; Zhu and Zhang, 2023; Zhao, 2026). An exploratory subgroup analysis was performed. The pooled RR was [I^2^ = 0%, fixed-effects model: RR = 1.33, 95% CI (1.11, 1.58), *P* = 0.002] for the 2-month subgroup and [I^2^ = 0%, fixed-effects model: RR = 1.24, 95% CI (1.11, 1.39), *P* = 0.0001] for the 3-month subgroup (Figure 2C). Both subgroups showed consistent beneficial effects. AF subtypes were heterogeneous across studies and could not be formally analyzed. Background therapy differed across all five studies, and no two studies used the same regimen; therefore, subgroup analysis by background therapy was not feasible.

Furthermore, leave-one-out sensitivity analyses consistently yielded similar estimates, with RRs ranging from 1.24 to 1.30, and all remained statistically significant [lowest: RR = 1.24, 95% CI (1.13, 1.39), *P* = 0.0001; highest: RR = 1.30, 95% CI (1.17, 1.46), *P* = 0.001] (Supplementary Figures S1.2–S1.6). Heterogeneity remained low in all leave-one-out analyses (I^2^ = 0%). These findings indicate that the pooled effect was robust and not driven by any single study.

##### 3.4.1.2 EHRA symptom score

Three studies reported the EHRA symptom scores (Zhang et al., 2019; Zhu and Zhang, 2023; Zhu and Zhang, 2024), a measure of AF symptom severity. Moderate heterogeneity was observed (I^2^ = 53%, *P* = 0.12). Using a random-effects model, the meta-analysis demonstrated that compared with the control group, YXDJ was associated with a statistically significant reduction in the EHRA symptom score [MD = −0.47, 95% CI (−0.74, −0.19), *P* = 0.0009], indicating a modest improvement in symptom burden (Figure 3A). This estimate represents postoperative patients only.

**FIGURE 3:**
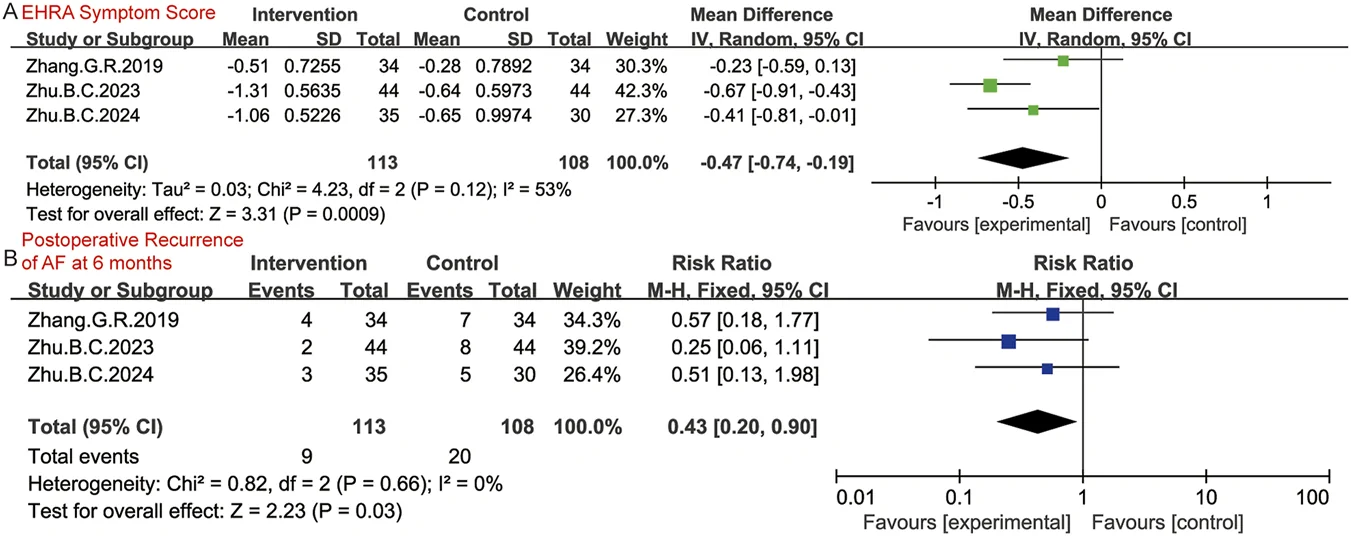
Forest Plots for the Effects of YXDJ on EHRA symptom score and postoperative recurrence rate of AF at 6 months. **(A,B)** Pooled forest plots for **(A)** the EHRA symptom score and **(B)** the postoperative recurrence rate of AF at 6 months.

We also examined other potential sources of clinical heterogeneity. All three studies were conducted in postoperative patients with a 3-month treatment duration; therefore, subgroup analyses by surgical status and treatment duration were not applicable. AF subtype, YXDJ dosage, and background therapy differed across studies, but their distribution was uneven and some categories contained only one study. Zhang GR (2019) (Zhang et al., 2019) enrolled patients with persistent and paroxysmal AF, used YXDJ 6.0 g/day, and background therapy was amiodarone plus warfarin. Zhu BC (2023) (Zhu and Zhang, 2023) enrolled patients with paroxysmal AF, used YXDJ 3.0 g/day, and background therapy was amiodarone without warfarin. Zhu BC (2024) (Zhu and Zhang, 2024) enrolled patients with paroxysmal AF, used YXDJ 8.0 g/day, and background therapy was antiarrhythmic drugs plus warfarin. Because these characteristics were unevenly distributed and some categories contained only one study, formal subgroup analyses were not feasible. Notably, Zhu BC (2023) (Zhu and Zhang, 2023) was the only study using a low dose of 3.0 g/day and the only one whose background therapy did not include warfarin; the other two studies used recommended doses (6–8 g/day) and background therapy including warfarin. In a sensitivity analysis excluding this study, heterogeneity decreased to 0% (*P* = 0.51) and yielded a consistent result [both fixed-effects and random-effects models: MD = −0.31, 95% CI (−0.58, −0.04), *P* = 0.02] (Supplementary Figures S2.1, S–2.2), suggesting that dosage and background therapy, in particular, may have contributed to the observed heterogeneity. AF subtype could not be analyzed separately, as all three studies included paroxysmal AF and only Zhang GR (2019) (Zhang et al., 2019) additionally enrolled patients with persistent AF.

#### 3.4.2 Postoperative recurrence of AF at 6 months

Three studies reported the AF recurrence at the 6-month postoperative time point (Zhang et al., 2019; Zhu and Zhang, 2023; Zhu and Zhang, 2024). As defined in the Methods, all three studies used a consistent definition of postoperative AF recurrence. All three studies detected recurrence primarily by 24-h Holter monitoring; none used event recorders or symptom-triggered ECG. Zhang GR (2019) (Zhang et al., 2019) and Zhu BC (2023) (Zhu and Zhang, 2023) performed 24-h Holter monitoring every 3 months, whereas Zhu BC (2024) (Zhu and Zhang, 2024) additionally used 12-lead ECG at 2 weeks, 3, 6, and 12 months after ablation. Although the monitoring schedule in Zhu BC (2024) (Zhu and Zhang, 2024) was slightly more intensive, the core detection method and recurrence definition were consistent across studies, and this minor difference is unlikely to have changed the overall direction of the effect.

No significant heterogeneity was detected (I^2^ = 0%, *P* = 0.66). A fixed-effects model meta-analysis showed that the YXDJ group had a lower risk of AF recurrence compared with the control group [RR = 0.43, 95% CI (0.20, 0.90), *P* = 0.03] (Figure 3B). A sensitivity analysis using a random-effects model yielded a consistent result [RR = 0.45, 95% CI (0.21, 0.95), *P* = 0.04] (Supplementary Figure S3.1).

The same clinical heterogeneity considerations apply as in the EHRA symptom score analysis, because the same three studies were included. A sensitivity analysis excluding Zhu BC (2023) (Zhu and Zhang, 2023) yielded a directionally consistent but no longer statistically significant result [I^2^ = 0%, fixed-effects model: RR = 0.55, 95% CI (0.23, 1.30), *P* = 0.17] (Supplementary Figure S3.2), suggesting that the pooled estimate was influenced by this study, although the direction of effect remained consistent. The loss of statistical significance after excluding Zhu BC (2023) (Zhu and Zhang, 2023) likely reflects the reduced number of studies and events, rather than a true difference in effect.

#### 3.4.3 Cardiac function

##### 3.4.3.1 LAEF

Four studies reported LAEF (Xue et al., 2020; Zhang et al., 2019; Zhu and Zhang, 2023; Zhu and Zhang, 2024). A random-effects meta-analysis showed that YXDJ was associated with improvements in LAEF compared with the control group [MD = 2.11%, 95% CI (0.70, 3.51), *P* = 0.003]. However, considerable heterogeneity was present (I^2^ = 71%, *P* = 0.02) (Figure 4A). This estimate mainly represents postoperative patients, as three of four studies were postoperative.

**FIGURE 4:**
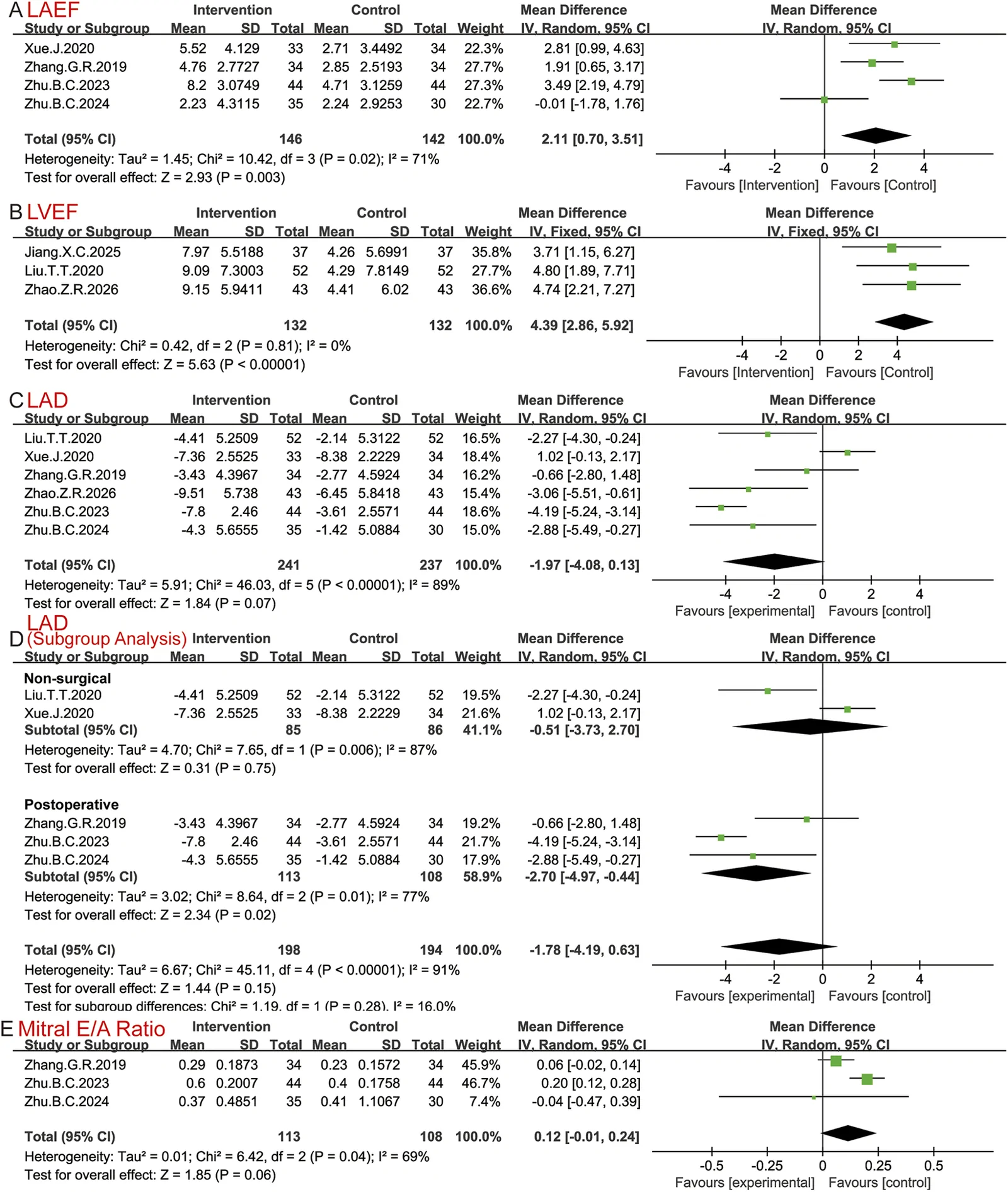
Forest Plots for the Effects of YXDJ on Cardiac Function. **(A–C)** Pooled forest plots for **(A)** LAEF, **(B)** LVEF, and **(C)** LAD. **(D)** Subgroup analysis of LAD by surgical status (postoperative vs. non-surgical). **(E)** Pooled forest plot for the mitral E/A ratio.

Sensitivity analysis indicated that the study by Zhu BC (2024) (Zhu and Zhang, 2024) was a major source of heterogeneity. Excluding this study substantially reduced heterogeneity (I^2^ = 32%, *P* = 0.23) while yielding a consistent and significant result using a random-effects model [MD = 2.71%, 95% CI (1.71, 3.72), *P* < 0.00001] (Supplementary Figure S4.1). After this exclusion, the remaining three studies were all conducted in postoperative patients, so this estimate represents the postoperative population more specifically.

We also examined other potential sources of clinical heterogeneity. Xue J (2020) (Xue et al., 2020) was the only non-surgical study and the only one with a 12-month treatment duration. Zhu BC (2023) (Zhu and Zhang, 2023) was the only study using 3.0 g/day and the only one whose background therapy did not include warfarin. In sensitivity analyses, excluding Xue J (2020) (Xue et al., 2020) yielded [I^2^ = 80%, random-effects model: MD = 1.88%, 95% CI (0.04, 3.71), *P* = 0.04] (Supplementary Figure S4.2), and excluding Zhu BC (2023) (Zhu and Zhang, 2023) yielded [I^2^ = 61%, random-effects model: MD = 1.59%, 95% CI (0.11, 3.07), *P* = 0.04] (Supplementary Figure S4.3). These sensitivity analyses showed that heterogeneity persisted even after excluding the non-surgical study or the low-dose study, indicating that statistical heterogeneity was not fully explained by these single factors. AF subtype could not be analyzed separately because all four studies included paroxysmal AF, and only Zhang GR (2019) (Zhang et al., 2019) additionally enrolled patients with persistent AF. After excluding Zhu BC (2024) (Zhu and Zhang, 2024), the remaining three studies were all postoperative, but they still differed in dosage and background therapy.

##### 3.4.3.2 LVEF

Three studies reported LVEF (Jiang and Lu, 2025; Liu et al., 2020; Zhao, 2026). No heterogeneity was observed (I^2^ = 0%, *P* = 0.81), so a fixed-effects model was used. The results showed that YXDJ was associated with improvements in LVEF compared with the control group [MD = 4.39%, 95% CI (2.86, 5.92), *P* < 0.00001] (Figure 4B). Sensitivity analysis using a random-effects model yielded consistent results [MD = 4.39%, 95% CI (2.86, 5.92), *P* < 0.00001] (Supplementary Figure S5.1). This estimate represents a mixed population of one postoperative and two non-surgical studies.

We also examined potential sources of clinical heterogeneity. All three studies used a treatment duration of approximately 3 months. Jiang XC (2025) (Jiang and Lu, 2025) was the only postoperative study and the only one using 3.0 g/day, whereas Liu TT (2020) (Liu et al., 2020) and Zhao ZR (2026) (Zhao, 2026) were both non-surgical and used 6.0 g/day; excluding Jiang XC (2025) (Jiang and Lu, 2025) yielded [I^2^ = 0%, fixed-effects model: MD = 4.77%, 95% CI (2.86, 6.67), *P* < 0.00001] (Supplementary Figure S5.2). This sensitivity analysis represents the non-surgical subgroup. Liu TT (2020) (Liu et al., 2020) was the only study enrolling patients with chronic heart failure and AF with a prolonged R-R interval, whereas the other two studies enrolled patients with paroxysmal AF; excluding it yielded [I^2^ = 0%, fixed-effects model: MD = 4.23%, 95% CI (2.43, 6.03), *P* < 0.00001] (Supplementary Figure S5.3). Background therapy differed across all three studies and could not be formally analyzed.

##### 3.4.3.3 LAD

Six studies provided data on LAD (Liu et al., 2020; Xue et al., 2020; Zhang et al., 2019; Zhu and Zhang, 2023; Zhu and Zhang, 2024; Zhao, 2026). Considerable heterogeneity was observed (I^2^ = 89%, *P* < 0.00001), prompting the use of a random-effects model. The overall pooled results demonstrated no statistically significant difference between groups [MD = −1.97 mm, 95% CI (−4.08, 0.13), *P* = 0.07] (Figure 4C).

To explore the sources of heterogeneity in LAD, we conducted a subgroup analysis stratified by prior cardiac surgery. Zhao ZR (2026) (Zhao, 2026) was excluded from this subgroup analysis because the surgical status of enrolled patients was not clearly defined in the original study (the decision to perform ablation was based on individual clinical judgment). In the postoperative subgroup, YXDJ was associated with a significant reduction in LAD [I^2^ = 77%, random-effects model: MD = −2.70 mm, 95% CI (−4.97, −0.44), *P* = 0.02]. In contrast, no significant effect was observed in the non-surgical subgroup [I^2^ = 87%, random-effects model: MD = −0.51 mm, 95% CI (−3.73, 2.70), *P* = 0.75] (Figure 4D). The non-surgical subgroup contained only two studies, limiting statistical power.

We then performed sensitivity analyses for treatment duration and dosage. Xue J (2020) (Xue et al., 2020) had a notably longer intervention duration (12 months vs. ≤ 3 months in the other studies); excluding this study reduced heterogeneity to I^2^ = 59% (*P* = 0.05) and yielded a statistically significant reduction in LAD favoring the YXDJ group [random-effects model: MD = −2.75 mm, 95% CI (−4.11, −1.39), *P* < 0.0001] (Supplementary Figure S6.1), although heterogeneity remained moderate. Zhu BC (2023) (Zhu and Zhang, 2023) was the only study using 3.0 g/day (vs. 6.0–8.0 g/day in the other studies); excluding this study yielded [I^2^ = 77%, random-effects model: MD = −1.40 mm, 95% CI (−3.23, 0.43), *P* = 0.13] (Supplementary Figure S6.2), suggesting that dosage may have contributed to the pooled estimate, but no definitive conclusion can be drawn. Liu TT (2020) (Liu et al., 2020) was the only study that did not enroll patients with paroxysmal AF (it enrolled patients with chronic heart failure and AF with a prolonged R-R interval); excluding it yielded [I^2^ = 91%, random-effects model: MD = −1.92 mm, 95% CI (−4.42, 0.58), *P* = 0.13] (Supplementary Figure S6.3). Background therapy differed across all six studies and could not be formally analyzed. Overall, the postoperative subgroup suggested a possible reduction in LAD, whereas the non-surgical subgroup showed no significant effect; however, these subgroup findings are limited by the small number of studies.

##### 3.4.3.4 Mitral E/A ratio

Three studies reported the mitral E/A ratio (Zhang et al., 2019; Zhu and Zhang, 2023; Zhu and Zhang, 2024), with substantial heterogeneity observed (I^2^ = 69%, *P* = 0.04), prompting the use of a random-effects model. The results showed that, compared with the control group, YXDJ did not lead to a statistically significant improvement in the mitral E/A ratio [MD = 0.12, 95% CI (−0.01, 0.24), *P* = 0.06] (Figure 4E). This estimate represents postoperative patients only.

The same clinical heterogeneity considerations apply as in the EHRA symptom score analysis. The heterogeneity was largely attributable to Zhu BC (2023) (Zhu and Zhang, 2023), which used a lower YXDJ dosage (3.0 g/day vs. 6.0–8.0 g/day in the other two studies) and whose background therapy did not include warfarin, unlike the other two studies. After excluding this study, heterogeneity decreased to 0% (*P* = 0.65), and the result remained directionally consistent but not statistically significant [MD = 0.06, 95% CI (−0.02, 0.14), *P* = 0.17] (Supplementary Figure S7.1). AF subtype could not be analyzed separately, as described in the EHRA symptom score analysis.

#### 3.4.4 Inflammatory biomarkers

##### 3.4.4.1 Hs-CRP

Five studies reported hs-CRP levels (Jiang and Lu, 2025; Xue et al., 2020; Zhang et al., 2019; Zhu and Zhang, 2023; Zhao, 2026), all five studies reported results in mg/L. Four studies specified immunoturbidimetry or an automated biochemical immunoassay based on immunoturbidimetry using venous blood samples (Xue et al., 2020; Zhang et al., 2019; Zhu and Zhang, 2023; Zhao, 2026), whereas one study did not report the detection method or sampling time (Jiang and Lu, 2025). Given the consistent units and largely comparable assays, the MD was used as the effect measure. Of these, three studies were conducted in postoperative patients (Jiang and Lu, 2025; Zhang et al., 2019; Zhu and Zhang, 2023) and two in non-surgical patients (Xue et al., 2020; Zhao, 2026). Therefore, the overall pooled estimate represents a mixed population of postoperative and non-surgical patients and should not be generalized to all AF populations. Moderate heterogeneity was observed (I^2^ = 60%, *P* = 0.04), and a random-effects model was therefore applied. The results indicated that YXDJ was associated with a statistically significant reduction in hs-CRP levels compared with the control group [random-effects model: MD = −1.21 mg/L, 95% CI (−1.63, −0.78), *P* < 0.00001] (Figure 5A).

**FIGURE 5:**
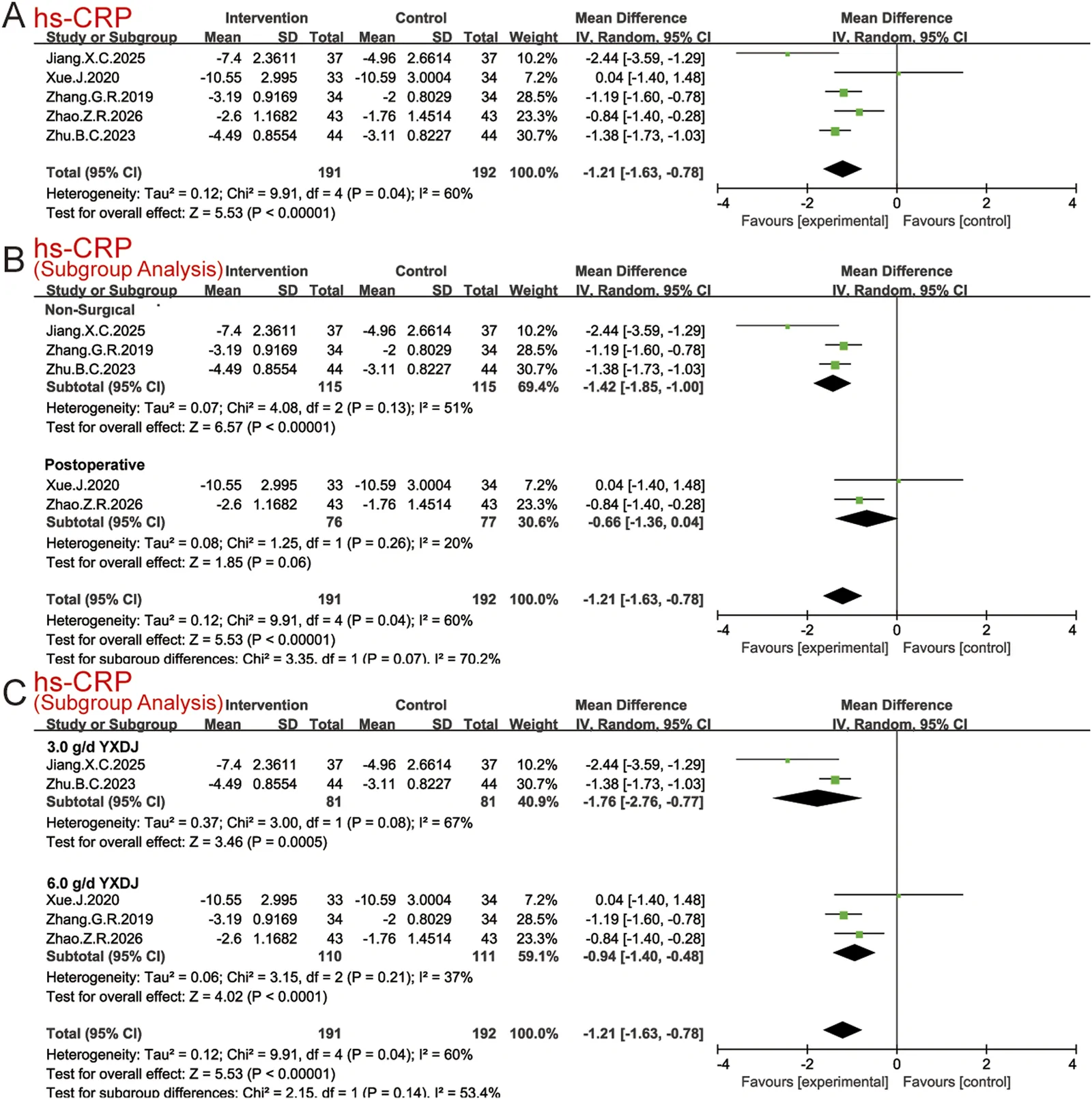
Forest Plots for the Effects of YXDJ on hs-CRP. **(A)** Pooled forest plot for hs-CRP. **(B)** Subgroup analysis of hs-CRP by surgical status (postoperative vs. non-surgical). **(C)** Subgroup analysis of hs-CRP by YXDJ dosage (3.0 g/d YXDJ vs. 6.0 g/d YXDJ).

Sensitivity analysis identified the study by Jiang XC (2025) (Jiang and Lu, 2025) as a source of heterogeneity. After excluding this study, heterogeneity decreased to I^2^ = 45% (*P* = 0.14), and the result remained consistent under a random-effects model [MD = −1.11 mg/L, 95% CI (−1.46, −0.75), *P* < 0.00001] (Supplementary Figure S8.1). However, the remaining four studies still included a mix of postoperative and non-surgical patients, so this estimate also represents a mixed population. This heterogeneity may be partly attributable to differences in detection methods or sampling time, because Jiang XC (2025) (Jiang and Lu, 2025) was the only study that did not report these details; however, other clinical differences, such as surgical status and YXDJ dosage, may also contribute.

We further examined potential sources of clinical heterogeneity through subgroup and sensitivity analyses. Subgroup analysis by prior cardiac surgery showed a significant reduction in hs-CRP in the postoperative subgroup [I^2^ = 51%, random-effects model: MD = −1.42 mg/L, 95% CI (−1.85, −1.00), *P* < 0.00001], whereas no statistically significant difference was observed in the non-surgical subgroup [I^2^ = 20%, MD = −0.66 mg/L, 95% CI (−1.36, 0.04), *P* = 0.06] (Figure 5B). An exploratory subgroup analysis by YXDJ dosage was also performed: two studies used 3.0 g/day (Jiang and Lu, 2025; Zhu and Zhang, 2023) and three used 6.0 g/day (Xue et al., 2020; Zhang et al., 2019; Zhao, 2026); the pooled MD was [I^2^ = 67%, random-effects model: MD = −1.76 mg/L, 95% CI (−2.76, −0.77), *P* = 0.0005] for the 3.0 g/day subgroup and [I^2^ = 37%, random-effects model: MD = −0.94 mg/L, 95% CI (−1.40, −0.48), *P* < 0.0001] for the 6.0 g/day subgroup (Figure 5C). For background therapy, Zhao ZR (2026) (Zhao, 2026) was the only study without amiodarone-based therapy (using sacubitril valsartan); excluding it yielded [I^2^ = 61%, random-effects model: MD = −1.32 mg/L, 95% CI (−1.82, −0.81), *P* < 0.00001] (Supplementary Figure S8.2). For treatment duration, Xue J (2020) (Xue et al., 2020) was the only study with a 12-month duration; excluding it yielded [I^2^ = 56%, random-effects model: MD = −1.29 mg/L, 95% CI (−1.68, −0.90), *P* < 0.00001] (Supplementary Figure S8.3). Zhang GR (2019) (Zhang et al., 2019) was the only study enrolling patients with persistent as well as paroxysmal AF, but formal analysis by AF subtype was not feasible because it was the only study in that category. Overall, these analyses showed that the direction of effect consistently favored YXDJ across subgroups and sensitivity analyses, although heterogeneity remained moderate in some analyses.

##### 3.4.4.2 TNF-α

Three studies reported TNF-α levels (Jiang and Lu, 2025; Zhang et al., 2019; Zhu and Zhang, 2023). Due to inconsistent units of measurement across studies, the SMD was selected as the effect measure. No statistical heterogeneity was observed (I^2^ = 0%, *P* = 0.46); therefore, a fixed-effects model was applied. To obtain a more conservative estimate, a random-effects model was also employed for sensitivity analysis, yielding consistent results. The analysis indicated that YXDJ was associated with a statistically significant reduction in TNF-α levels compared with the control group, with a moderate effect size [both fixed- and random-effects models: SMD = −0.44, 95% CI (−0.71, −0.18), *P* = 0.0009] (Figure 6A; Supplementary Figure S9.1). This estimate represents postoperative patients only.

**FIGURE 6:**
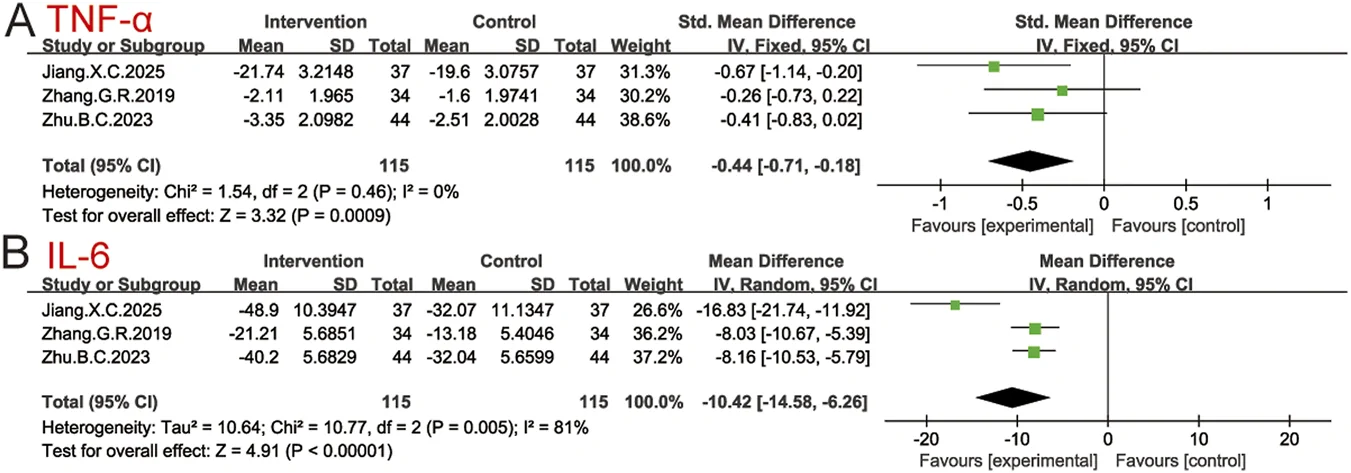
Forest Plots for the Effects of YXDJ on TNF-α and IL-6. **(A,B)** Pooled forest plots for **(A)** TNF-α and **(B)** IL-6.

All three studies were conducted in postoperative patients with a 3-month treatment duration. Zhang GR (2019) (Zhang et al., 2019) differed from the other two studies in that it used a higher dose of 6.0 g/day (vs. 3.0 g/day), a background therapy that included warfarin (vs. amiodarone alone), and enrolled patients with persistent as well as paroxysmal AF (vs. paroxysmal AF alone). A sensitivity analysis excluding this study yielded [I^2^ = 0%, fixed-effects model: SMD = −0.53, 95% CI (−0.84, −0.21), *P* = 0.001] (Supplementary Figure S9.2), indicating that the pooled estimate was not driven by this single study.

##### 3.4.4.3 IL-6

Three studies reported IL-6 levels (Jiang and Lu, 2025; Zhang et al., 2019; Zhu and Zhang, 2023). All three studies reported results in ng/L. Two studies specified a colorimetric assay using fasting venous blood samples (Zhang et al., 2019; Zhu and Zhang, 2023), whereas one study did not report the detection method or sampling time (Jiang and Lu, 2025). Given the consistent units and largely comparable assays, the MD was used as the effect measure. Substantial heterogeneity was observed (I^2^ = 81%, *P* = 0.005), and a random-effects model was therefore applied. The initial pooled results indicated that YXDJ was associated with a statistically significant reduction in IL-6 levels compared with the control group [MD = −10.42 ng/L, 95% CI (−14.58, −6.26), *P* < 0.00001] (Figure 6B). This estimate represents postoperative patients only.

Given the substantial heterogeneity, we performed leave-one-out sensitivity analyses. Exclusion of Jiang XC (2025) (Jiang and Lu, 2025) reduced heterogeneity to 0% (*P* = 0.94) and yielded a consistent effect estimate [MD = −8.10 ng/L, 95% CI (−9.86, −6.34), *P* < 0.00001] (Supplementary Figure S10.1), indicating that this study was the main source of statistical heterogeneity. This heterogeneity may be partly attributable to differences in detection methods or sampling time, because Jiang XC (2025) (Jiang and Lu, 2025) was the only study that did not report these details; however, other clinical differences cannot be excluded.

From a clinical perspective, as in the TNF-α analysis, Zhang GR (2019) (Zhang et al., 2019) was the only study with 6.0 g/day, warfarin, and persistent and paroxysmal AF. However, excluding this study yielded [I^2^ = 90%, random-effects model: MD = −12.22 ng/L, 95% CI (−20.70, −3.74), *P* = 0.005], with heterogeneity remaining high (Supplementary Figure S10.2). This suggests that the heterogeneity was primarily attributable to Jiang XC (2025) (Jiang and Lu, 2025), rather than to the clinical differences examined. Because only two studies remained in each sensitivity analysis, these results should be considered exploratory.

### 3.5 Adverse reactions

Six studies reported safety outcomes (Jiang and Lu, 2025; Liu et al., 2020; Xue et al., 2020; Zhang et al., 2020; Zhu and Zhang, 2023; Zhao, 2026) (Table 2). A fixed-effects meta-analysis (I^2^ = 0%, *P* = 0.43) found no statistically significant difference in the overall risk of adverse events between the YXDJ group and the control group [RR = 0.58, 95% CI (0.32, 1.04), *P* = 0.07] (Figure 7A). Sensitivity analysis using a random-effects model yielded consistent results [RR = 0.68, 95% CI (0.36, 1.26), *P* = 0.22] (Supplementary Figure S11.1). Postoperative and non-surgical subgroups were analyzed separately.

**TABLE 2:** Adverse events in the combined YXDJ group and control group.

Adverse event type	YXDJ group (n/N, %)	Control group (n/N, %)	Notes	Studies involved
Gastrointestinal disorders	9/259 (3.5%)	11/269 (4.1%)	Nausea, vomiting, diarrhea, loss of appetite	(Jiang and Lu, 2025; Liu et al., 2020; Xue et al., 2020; Zhang et al., 2020; Zhu and Zhang, 2023; Zhao, 2026)
Nervous system disorders	4/216 (1.9%)	4/226 (1.8%)	Dizziness, amaurosis, headache	Jiang and Lu, 2025; Liu et al., 2020; Zhang et al., 2020; Zhu and Zhang, 2023)
Skin and subcutaneous tissue disorders	1/216 (0.5%)	1/226 (0.4%)	Rash	(Jiang and Lu, 2025)
Cardiac disorders	0/216 (0.0%)	9/226 (4.0%)	Sinus bradycardia, atrioventricular block	(Xue et al., 2020)
Hepatic disorders	1/259 (0.4%)	1/269 (0.4%)	Abnormal liver function	(Zhao, 2026)
Vascular disease	1/259 (0.4%)	3/269 (1.1%)	Angioedema, hypotension	(Zhao, 2026)
Total	16/259 (6.2%)	29/269 (10.8%)	​	​

Total refers to the number of patients experiencing at least one adverse event. Denominators differ across categories because not all studies reported every type of adverse event.

**FIGURE 7:**
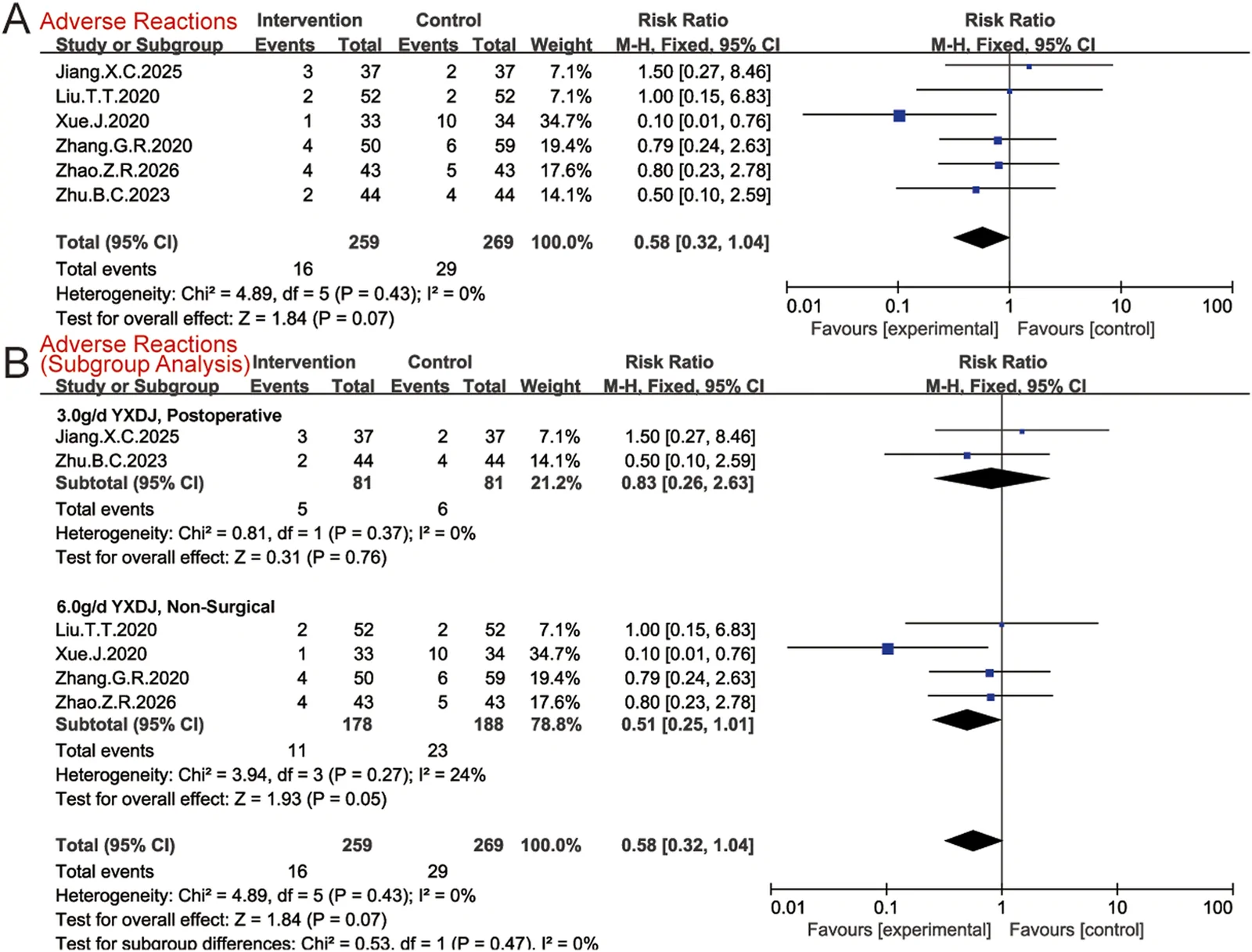
Forest Plots for the Effects of YXDJ on Adverse Reactions. **(A)** Pooled forest plots for adverse reactions. **(B)** Subgroup analysis of adverse reactions by surgical status/YXDJ dosage (postoperative/3.0 g/d YXDJ vs. non-surgical/6.0 g/d YXDJ).

We also examined potential sources of clinical heterogeneity. Subgroup analyses by surgical status and YXDJ dosage were performed, but the postoperative and 3.0 g/day subgroups both contained the same two studies (Jiang and Lu, 2025; Zhu and Zhang, 2023). The postoperative/3.0 g/day subgroup estimate represents postoperative patients receiving low-dose YXDJ, whereas the non-surgical/6.0 g/day subgroup estimate represents non-surgical patients receiving the recommended dose. The pooled RR was [I^2^ = 0%, fixed-effects model: RR = 0.83, 95% CI (0.26, 2.63), *P* = 0.76] for the postoperative/3.0 g/day subgroup (Jiang and Lu, 2025; Zhu and Zhang, 2023) and [I^2^ = 24%, fixed-effects model: RR = 0.51, 95% CI (0.25, 1.01), *P* = 0.05] for the non-surgical/6.0 g/day subgroup (Liu et al., 2020; Xue et al., 2020; Zhang et al., 2020; Zhao, 2026) (Figure 7B). Liu TT (2020) (Liu et al., 2020) was the only study enrolling patients with chronic heart failure and AF with a prolonged R-R interval, whereas the other five studies enrolled patients with paroxysmal AF; excluding it yielded [I^2^ = 15%, fixed-effects model: RR = 0.54, 95% CI (0.29, 1.01), *P* = 0.05] (Supplementary Figure S11.2). For treatment duration, Xue J (2020) (Xue et al., 2020) had a 12-month duration and Zhang GR (2020) (Zhang et al., 2020) had a 1-month duration, whereas the other four studies used approximately 3 months; excluding these two studies yielded [I^2^ = 0%, fixed-effects model: RR = 0.85, 95% CI (0.39, 1.84), *P* = 0.67] (Supplementary Figure S11.3). Background therapy differed across all six studies and could not be formally analyzed.

### 3.6 Long-term follow-up and other exploratory outcomes

Several additional outcomes were reported in only two studies and therefore could not be meta-analyzed (Table 3). Given the limited number of studies and the small sample sizes, these findings should be considered exploratory.

**TABLE 3:** Matrix of outcome availability across included studies.

Author (Year)	Antiarrhythmic efficacy	Electrophysiological parameters	Cardiac structure and function	Inflammation and oxidative stress	Neuroendocrine activation and hemodynamic load	Safety and clinical endpoints
(Jiang and Lu, 2025)	TCM Syndrome Score	-	LVEDV, LVESV, LVEF, Stroke Volume	hs-CRP, TNF-α, IL-6	-	Adverse Reactions
(Liu et al., 2020)	Overall Clinical Response Rate	Heart Rate, SDNN, SDANN, RMSSD, PNN50, Total Heartbeats (in 24 h), Longest RR Interval, Number of Long RR Intervals	LAD, LVEDD, LVEF	-	NT-proBNP	Adverse Reactions
(Xiao and Sun, 2020)	Overall Clinical Response Rate	LAERP	-	-	-	-
(Xue et al., 2020)	Sinus Rhythm Maintenance	Heart Rate, P Wave Dispersion	LAD, LAEDV, LAESV, LAEF	hs-CRP	Systolic Blood Pressure, Diastolic Blood Pressure	Adverse Reactions
(Ying et al., 2021)	Overall Clinical Response Rate, Frequency of AF Recurrence and Cardioversion	-	LARV, LACV, LAAEF, A Peak	Reactive Oxygen Species, AOPP	-	-
(Zhang et al., 2020)	-	-	-	-	-	Adverse Reactions, Cerebrovascular Events
(Zhang et al., 2019)	Postoperative Recurrence of AF, EHRA Symptom Score, SF-36	-	LAD, LAEF, Mitral E/A ratio	hs-CRP, TNF-α, IL-6	-	-
(Zhu and Zhang, 2023)	Overall Clinical Response Rate, Postoperative Recurrence of AF, EHRA Symptom Score, SF-36	-	LAD, LAEDV, LAESV, LAEF, Mitral E/A ratio	hs-CRP, TNF-α, IL-6	-	Adverse Reactions
(Zhu and Zhang, 2024)	Postoperative Recurrence of AF, EHRA Symptom Score	-	LAD, LAEF, Mitral E/A ratio	-	-	-
(Zhao, 2026)	Overall Clinical Response Rate	-	LAD, LVEF	hs-CRP	BNP, Angiotensin II	Adverse Reactions

“-” indicates that the outcome was not reported in the original study. Abbreviations: AOPP, advanced oxidation protein products; BNP, B-type Natriuretic Peptide; EHRA, european heart rhythm association score; hs-CRP, High-Sensitivity C-Reactive Protein; IL-6, Interleukin-6; LAAEF, left atrial active ejection fraction; LACV, left atrial conduit volume; LAD, left atrial diameter; LAEDV, Left Atrial End-Diastolic Volume; LAEF, left atrial ejection fraction; LAERP, left atrial effective refractory period; LAESV, Left Atrial End-Systolic Volume; LARV, left atrial reservoir volume; LVEDD, Left Ventricular End-Diastolic Diameter; LVEDV, Left Ventricular End-Diastolic Volume; LVEF, left ventricular ejection fraction; LVESV, Left Ventricular End-Systolic Volume; Mitral E/A ratio, Ratio of Early (E) to Late (A) mitral inflow velocity; NT-proBNP, N-Terminal Pro-B-Type Natriuretic Peptide; PNN50, Percentage of NN50; RMSSD, root mean square of successive differences; SDANN, Standard Deviation of Average NN, intervals; SDNN, Standard Deviation of NN, intervals; SF-36, 36-Item Short Form Health Survey; TCM, syndrome score, Traditional Chinese Medicine Syndrome Score; TNF-α, Tumor Necrosis Factor-Alpha.

#### 3.6.1 Long-term prognostic outcomes

Ischemic stroke: One single-center study specifically designed to evaluate the preventive effect of YXDJ on ischemic stroke in patients with non-valvular atrial fibrillation reported outcomes during a mean follow-up of 10.7 ± 2.4 months (Zhang et al., 2020). During this extended observation window, 3 of 50 patients (6.00%) in the YXDJ group experienced ischemic stroke, compared with 5 of 59 patients (8.47%) in the control group. The original authors reported that YXDJ was associated with a lower risk of ischemic stroke in univariate analysis (HR = 0.39, 95% CI 0.17–0.93, *P* = 0.023) and in a multivariable model (HR = 0.45, 95% CI 0.15–0.97, *P* = 0.045). However, the covariates included in the multivariable model were not reported, and only eight stroke events occurred, making the multivariable estimate unstable. Therefore, this finding should be regarded as exploratory and hypothesis-generating, and it should not be interpreted as evidence of an independent cerebrovascular protective effect beyond conventional anticoagulation.

Postoperative outcomes at 9 months: Two studies extended follow-up to 9 months post-ablation for recurrence rates and symptom scores (Zhang et al., 2019; Zhu and Zhang, 2024). At the 9-month follow-up, both studies consistently demonstrated that the YXDJ group had a significantly lower AF recurrence rate compared with the control group: Zhang GR et al. (2019) reported 7/34 (20.58%) vs. 10/34 (29.41%) (Zhang et al., 2019), and Zhu BC et al. (2024) reported 7/35 (20.00%) vs. 11/30 (36.67%) (Zhu and Zhang, 2024). Both studies also reported significantly improved EHRA symptom scores at 9 months favoring the YXDJ group. However, echocardiographic parameters (LAD, LAEF, E/A) were only reported up to 6 months in Zhang GR et al. (Zhang et al., 2019), while Zhu BC et al. (Zhu and Zhang, 2024) extended cardiac function assessments to 9 months. These extended follow-up data suggest that the therapeutic benefits of YXDJ on rhythm control and symptom relief may be sustained beyond the initial 6-month post-ablation period. However, these data are derived from only two studies and should be considered exploratory.

#### 3.6.2 Other exploratory outcomes

Sinus rhythm maintenance rate: One study reported the sinus rhythm maintenance rate at 3, 6, and 12 months in patients with paroxysmal AF (Xue et al., 2020). No significant difference was observed between the YXDJ and control groups at any time point (*P* > 0.05 for all comparisons).

Frequency of AF recurrence and cardioversion: One study reported the number of AF recurrences and pharmacologic cardioversions in patients with hypertension and paroxysmal AF (Ying et al., 2021). The YXDJ group had significantly fewer AF recurrences than the control group [median (IQR): 5 (3, 6) vs. 6 (4, 6), *P* = 0.004], while no significant difference was observed in the number of pharmacologic cardioversions between the two groups [median (IQR): 2 (1, 3) vs. 2 (1, 3), *P* = 0.459].

Left atrial end-diastolic volume (LAEDV) and left atrial end-systolic volume (LAESV): Two studies reported LAEDV and LAESV in AF patients (Xue et al., 2020; Zhu and Zhang, 2023). Both studies observed that YXDJ combined with conventional therapy significantly reduced LAEDV and LAESV compared with conventional therapy alone (all *P* < 0.05), suggesting a potential benefit in attenuating left atrial volume overload.

Short Form-36 Health Survey (SF-36) quality of life score: Two studies reported SF-36 scores (Zhang et al., 2019; Zhu and Zhang, 2023). Zhu BC et al. (2023) showed that YXDJ significantly improved the total SF-36 score compared with the control group (*P* < 0.05). Zhang GR (2019) reported significant improvements in the physical functioning and mental health domains (all *P* < 0.05). These findings suggest potential benefits in health-related quality of life.

Heart rate: Two studies reported changes in heart rate before and after treatment (Liu et al., 2020; Xue et al., 2020). In Liu TT (2020), both the YXDJ and control groups showed a reduction in heart rate from approximately 80 bpm to 72 bpm, with no significant difference between the two groups either at baseline or after treatment. Similarly, in Xue J (2020), both groups demonstrated a decrease from approximately 120 bpm to 85–90 bpm, also with no significant between-group difference at baseline or post-treatment. These findings suggest that YXDJ did not affect heart rate beyond the effects of conventional therapy alone.

B-type natriuretic peptide (BNP) /N-terminal pro-B-type natriuretic peptide (NT-proBNP): Two studies reported BNP or NT-proBNP levels (Liu et al., 2020; Zhao, 2026). Liu TT (2020) measured NT-proBNP in patients with AF and chronic heart failure and found that YXDJ combined with conventional therapy significantly reduced NT-proBNP levels compared with conventional therapy alone (*P* < 0.05) (Liu et al., 2020). Similarly, Zhao ZR (2026) measured BNP in patients with paroxysmal AF and reported that the YXDJ group showed significantly greater reduction in BNP levels than the control group (*P* < 0.05) (Zhao, 2026).

### 3.7 Assessment of publication bias

Formal statistical assessment of publication bias was not performed because each outcome included fewer than 10 studies, as recommended by the Cochrane Handbook. Funnel plots were not generated for any endpoint. Therefore, publication bias could not be assessed. Given that all included studies were small, Chinese-language, single-center trials, the risk of publication bias cannot be excluded.

### 3.8 GRADE evidence profile

A summary of all primary, subgroup, and sensitivity analyses is presented in Table 4. The overall clinical response rate and EHRA symptom score were rated as very low-quality evidence due to serious risk of bias (lack of blinding) and indirectness (subjective composite endpoints). Although the RoB 2.0 assessment rated all included RCTs as “Some concerns,” we considered the risk of bias for these subjective outcomes to be more severe because the lack of blinding of outcome assessors could substantially influence subjective measurements; therefore, we downgraded these outcomes by two levels. The 6-month AF recurrence rate was rated as moderate-quality evidence—downgraded for risk of bias and imprecision (wide 95% CI) but upgraded for the large effect size (RR = 0.43). Cardiac function parameters (LAEF, LAD) were rated as low-quality evidence, primarily due to some concerns in risk of bias (mainly from randomization process, deviations from intended interventions, and selective reporting) and moderate-to-substantial heterogeneity across studies. In contrast, LVEF was rated as moderate-quality evidence, given its objective nature, consistent effect across studies (I^2^ = 0%), and robust statistical significance. Inflammatory biomarkers (hs-CRP, TNF-α, IL-6) were rated as low-quality evidence, mainly due to some concerns in risk of bias and, in some cases, inconsistency. The overall incidence of adverse events was rated as low-quality evidence due to risk of bias and imprecision. No outcomes were rated as high quality (Table 5).

**TABLE 4:** Summary of meta-analysis, subgroup, and sensitivity analysis results.

Outcome	Analysis	Population represented	n	I^2^	Model	Effect estimate (RR/MD)	95% CI	*P*
Overall clinical response rate								
​	Main analysis	Mixed	5	0%	Fixed	RR = 1.27	(1.16, 1.40)	<0.00001
Sensitivity	Main analysis	Mixed	5	0%	Random	RR = 1.26	(1.15, 1.39)	<0.00001
Subgroup 1	symptom-based^a^	Non-surgical	3	0%	Fixed	RR = 1.27	(1.12, 1.45)	0.0003
​	objective-based^a^	Mixed	2	0%	Fixed	RR = 1.28	(1.12, 1.47)	0.0004
Subgroup 2	2-month duration^e^	Non-surgical	2	0%	Fixed	RR = 1.33	(1.11, 1.58)	0.002
​	3-month duration^e^	Mixed	3	0%	Fixed	RR = 1.24	(1.11, 1.39)	0.0001
Sensitivity	exclude Zhu BC (2023)^b^ ^,^ ^c^ ^,^ ^g^	Non-surgical	4	0%	Fixed	RR = 1.26	(1.13, 1.40)	<0.0001
Sensitivity	exclude Liu TT (2020)^g^	Mixed	4	0%	Fixed	RR = 1.30	(1.17, 1.46)	<0.0001
Sensitivity	exclude Xiao KH (2020)^g^	Mixed	4	0%	Fixed	RR = 1.27	(1.15, 1.41)	<0.00001
Sensitivity	exclude Ying LH (2021)^g^	Mixed	4	0%	Fixed	RR = 1.25	(1.13, 1.39)	<0.0001
Sensitivity	exclude Zhao ZR (2026)^g^	Mixed	4	0%	Fixed	RR = 1.29	(1.16, 1.44)	<0.0001
EHRA symptom score								
​	Main analysis	Postoperative	3	53%	Random	MD = −0.47	(-0.74, −0.19)	0.0009
Sensitivity	exclude Zhu BC (2023)^c^ ^,^ ^d^	Postoperative	2	0%	Fixed	MD = −0.31	(-0.58, −0.04)	0.02
Sensitivity	exclude Zhu BC (2023)^c^ ^,^ ^d^	Postoperative	2	0%	Random	MD = −0.31	(-0.58, −0.04)	0.02
Postoperative recurrence of AF at 6 months								
​	Main analysis	Postoperative	3	0%	Fixed	RR = 0.43	(0.20, 0.90)	0.03
Sensitivity	Main analysis	Postoperative	3	0%	Random	RR = 0.45	(0.21, 0.95)	0.04
Sensitivity	exclude Zhu BC (2023)^c^ ^,^ ^d^	Postoperative	2	0%	Fixed	RR = 0.55	(0.23, 1.30)	0.17
LAEF								
​	Main analysis	Mixed	4	71%	Random	MD = 2.11%	(0.70, 3.51)	0.003
Sensitivity	exclude Zhu BC (2024)^g^	Postoperative	3	32%	Random	MD = 2.71%	(1.71, 3.72)	<0.00001
Sensitivity	exclude Xue J (2020)^b^ ^,^ ^e^	Postoperative	3	80%	Random	MD = 1.88%	(0.04, 3.71)	0.04
Sensitivity	exclude Zhu BC (2023)^c^ ^,^ ^d^	Mixed	3	61%	Random	MD = 1.59%	(0.11, 3.07)	0.04
LVEF								
​	Main analysis	Mixed	3	0%	Fixed	MD = 4.39%	(2.86, 5.92)	<0.00001
Sensitivity	Main analysis	Mixed	3	0%	Random	MD = 4.39%	(2.86, 5.92)	<0.00001
Sensitivity	exclude Jiang XC (2025)^b^ ^,^ ^c^	Non-surgical	2	0%	Fixed	MD = 4.77%	(2.86, 6.67)	<0.00001
Sensitivity	exclude Liu TT (2020)^f^	Mixed	2	0%	Fixed	MD = 4.23%	(2.43, 6.03)	<0.00001
LAD								
​	Main analysis	Mixed	6	89%	Random	MD = −1.97 mm	(-4.08, 0.13)	0.07
Subgroup exclude Zhao ZR (2026)	postoperative^b^	Postoperative	3	77%	Random	MD = −2.70 mm	(-4.97, −0.44)	0.02
​	non-surgical^b^	Non-surgical	2	87%	Random	MD = −0.51 mm	(-3.73, 2.70)	0.75
Sensitivity	exclude Xue J (2020)^e^	Mixed	5	59%	Random	MD = −2.75 mm	(-4.11, −1.39)	<0.0001
Sensitivity	exclude Zhu BC (2023)^c^	Mixed	5	77%	Random	MD = −1.40 mm	(-3.23, 0.43)	0.13
Sensitivity	exclude Liu TT (2020)^f^	Mixed	5	91%	Random	MD = −1.92 mm	(-4.42, 0.58)	0.13
Mitral E/A ratio								
​	Main analysis	Postoperative	3	69%	Random	MD = 0.12	(-0.01, 0.24)	0.06
Sensitivity	exclude Zhu BC (2023)^c^ ^,^ ^d^	Postoperative	2	0%	Random	MD = 0.06	(-0.02, 0.14)	0.17
hs-CRP								
​	Main analysis	Mixed	5	60%	Random	MD = −1.21 mg/L	(-1.63, −0.78)	<0.00001
Subgroup 1	postoperative^b^	Postoperative	3	51%	Random	MD = −1.42 mg/L	(-1.85, −1.00)	<0.00001
​	non-surgical^b^	Non-surgical	2	20%	Random	MD = −0.66 mg/L	(-1.36, 0.04)	0.06
Subgroup 2	3.0 g/d^c^	Postoperative	2	67%	Random	MD = −1.76 mg/L	(-2.76, −0.77)	0.0005
​	6.0 g/d^c^	Mixed	3	37%	Random	MD = −0.94 mg/L	(-1.40, −0.48)	<0.0001
Sensitivity	exclude Jiang XC (2025)^g^	Mixed	4	45%	Random	MD = −1.11 mg/L	(-1.46, −0.75)	<0.00001
Sensitivity	exclude Zhao ZR (2026)^d^	Mixed	4	61%	Random	MD = −1.32 mg/L	(-1.82, −0.81)	<0.00001
Sensitivity	exclude Xue J (2020)^e^	Mixed	4	56%	Random	MD = −1.29 mg/L	(-1.68, −0.90)	<0.00001
TNF-α								
​	Main analysis	Postoperative	3	0%	Fixed	SMD = −0.44	(-0.71, −0.18)	0.0009
Sensitivity	Main analysis	Postoperative	3	0%	Random	SMD = −0.44	(-0.71, −0.18)	0.0009
Sensitivity	exclude Zhang GR (2019)^c^ ^,^ ^d^ ^,^ ^f^	Postoperative	2	0%	Fixed	SMD = −0.53	(-0.84, −0.21)	0.001
IL-6								
​	Main analysis	Postoperative	3	81%	Random	MD = −10.42 ng/L	(-14.58, −6.26)	<0.00001
Sensitivity	exclude Jiang XC (2025)^g^	Postoperative	2	0%	Random	MD = −8.10 ng/L	(-9.86, −6.34)	<0.00001
Sensitivity	exclude Zhang GR (2019)^c^ ^,^ ^d^ ^,^ ^f^	Postoperative	2	90%	Random	MD = −12.22 ng/L	(-20.70, −3.74)	0.005
Adverse reactions								
​	Main analysis	Mixed	6	0%	Fixed	RR = 0.58	(0.32, 1.04)	0.07
Sensitivity	Main analysis	Mixed	6	0%	Random	RR = 0.68	(0.36, 1.26)	0.22
Subgroup	postoperative/3.0 g/d^b^ ^,^ ^c^	Postoperative	2	0%	Fixed	RR = 0.83	(0.26, 2.63)	0.76
​	non-surgical/6.0 g/d^b^ ^,^ ^c^	Non-surgical	4	24%	Fixed	RR = 0.51	(0.25, 1.01)	0.05
Sensitivity	exclude Liu TT (2020)^f^	Mixed	5	15%	Fixed	RR = 0.54	(0.29, 1.01)	0.05
Sensitivity	exclude Xue J (2020) + Zhang GR (2020)^e^	Mixed	4	0%	Fixed	RR = 0.85	(0.39, 1.84)	0.67

RR, risk ratio; MD, mean difference; SMD, standardized mean difference; CI, confidence interval; I^2^.

heterogeneity; n, number of studies included in the analysis. Reasons for subgroup and sensitivity analyses.

^a^ Definition type of overall clinical response rate (symptom-based vs. incorporating objective cardiac assessments);

^b^ Surgical status (postoperative vs. non-surgical);

^c^ YXDJ, dosage (3.0 g/day vs. 6.0–8.0 g/day);

^d^ Background therapy;

^e^ Treatment duration;

^f^ AF, subtype/underlying condition;

^g^ Leave-one-out sensitivity analysis.

**TABLE 5:** GRADE evidence profile for key outcomes.

Outcome	Studies	Risk of bias	Inconsistency	Indirectness	Imprecision	Publication bias	Upgrading	Quality	Reasons for downgrade/upgrade
Overall Clinical Response Rate	5	Serious (−2)	No	Serious (−1)	No	No	No	⊕○○○ Very low	Lack of blinding for subjective outcomes, despite overall RoB 2.0 rating of “Some concerns”; non-uniform diagnostic criteria across trials
EHRA Symptom Score	3	Serious (−2)	No	No	Serious (−1)	No	No	⊕○○○ Very low	Patient-reported subjective outcome; no blinding in most studies; small sample size
Postoperative Recurrence of AF at 6 Months	3	Serious (−1)	No	No	Serious (−1)	No	Large effect (+1)	⊕⊕⊕○ Moderate	Lack of blinding, though recurrence was objectively confirmed by ECG/Holter; relatively wide 95% CI (0.20–0.90) based on only 3 studies; RR = 0.43 qualifies as large effect
LAEF	4	Serious (−1)	Serious (−1)	No	No	No	No	⊕⊕○○ Low	Some concerns in RoB 2.0 (randomization process, deviations, and selective reporting); moderate heterogeneity (I^2^ = 71%); sensitivity analysis confirmed robustness
LVEF	3	Serious (−1)	No	No	No	No	No	⊕⊕⊕○ Moderate	Some concerns in RoB 2.0 (randomization process, deviations, and selective reporting); LVEF is a well-established objective parameter, and results were highly consistent (I^2^ = 0%)
LAD (Overall)	6	Serious (−1)	Very serious (−2)	No	No	No	No	⊕○○○ Very low	High heterogeneity (I^2^ = 89%); result not statistically significant (P = 0.07); driven by one study with longer duration
LAD (Postop Subgroup)	3	Serious(-1)	Serious (−1)	No	No	No	No	⊕⊕○○ Low	Moderate heterogeneity (I^2^ = 77%) but significant result (P = 0.02); clinically meaningful in postoperative patients
Mitral E/A Ratio	3	Serious (−1)	Serious (−1)	No	Serious (−1)	No	No	⊕○○○ Very low	Some concerns in RoB 2.0; high heterogeneity (I^2^ = 69%); non-significant (P = 0.06); small sample size; one study used lower YXDJ dose
hs-CRP (Overall)	5	Serious (−1)	Serious (−1)	No	No	No	No	⊕⊕○○ Low	Some concerns in RoB 2.0; moderate heterogeneity (I^2^ = 60%); consistent direction of effect
hs-CRP (Postop Subgroup)	3	Serious (−1)	Serious (−1)	No	No	No	No	⊕⊕○○ Low	Moderate heterogeneity (I^2^ = 51%); larger effect in postoperative patients (MD = −1.42 vs. −1.21); consistent direction
TNF-α	3	Serious (−1)	No	Serious (−1)	No	No	No	⊕⊕○○ Low	Inconsistent units necessitated SMD (indirectness); consistent effect (I^2^ = 0%); some concerns in RoB 2.0
IL-6	3	Serious (−1)	Serious (−1)	No	No	No	No	⊕⊕○○ Low	High heterogeneity (I^2^ = 81%); driven by one study; sensitivity analysis confirmed robustness
Adverse Reactions	6	Serious (−1)	No	No	Serious (−1)	No	No	⊕⊕○○ Low	95% CI crosses 1 (0.32–1.04); cannot rule out no difference; small number of events; selective reporting possible

## 4 Discussion

The search for optimal AF management remains challenging because of the limited efficacy and tolerability of current therapies, which has driven interest in complementary TCM approaches. Several standardized Chinese patent medicines with *qi*- and *yin*-supplementing properties, including Shensong Yangxin Capsule, Wenxin Granules, and YXDJ, have been used as adjunctive therapies for AF. Among these, Shensong Yangxin Capsule (Shi et al., 2021; Chen et al., 2025) and Wenxin Granules (Wang et al., 2022; Qi et al., 2024) have been extensively investigated in meta-analyses that synthesized evidence from numerous RCTs and supported their beneficial effects. Although YXDJ shares a similar therapeutic principle, it places greater emphasis on nourishing yin and tonifying blood, and an increasing number of recent RCTs have reported its potential efficacy in AF. However, the existing meta-analysis on YXDJ included heterogeneous arrhythmia populations rather than focusing specifically on AF (Wang XY. et al., 2021), leaving the accumulating AF-specific evidence unsynthesized.

Pooling data from 10 RCTs, our findings suggest that YXDJ combined with conventional therapy may confer additional benefits in improving clinical symptoms, reducing postoperative recurrence, alleviating inflammation, and promoting recovery of certain cardiac functions, with no clear evidence of an increased risk of adverse events. However, the overall quality of evidence ranged from low to very low for most outcomes, with only LVEF and 6-month postoperative AF recurrence rated as moderate certainty. These findings should therefore be considered preliminary and hypothesis-generating. In addition, all included RCTs were conducted in China, and the enrolled patients were predominantly of East Asian descent; none reported detailed racial or ethnic composition. Ethnicity may influence AF epidemiology, drug metabolism, anticoagulation response, inflammatory profiles, and responses to traditional Chinese medicine. Therefore, the findings may not be directly generalizable to non-East Asian populations, and the role of ethnicity as an effect modifier could not be assessed.

### 4.1 Symptom improvement

YXDJ was associated with improvements in the overall clinical response rate compared with conventional therapy alone [RR = 1.27, 95% CI (1.16, 1.40), *P* < 0.00001]. The lower bound of the 95% CI (1.16) suggests that the true benefit could be as small as a 16% relative increase in response, whereas the upper bound (1.40) suggests it could be as large as a 40% increase. This is a modest-to-moderate effect, but the outcome is a subjective, study-defined composite endpoint that lacks uniform diagnostic criteria across trials and is susceptible to detection bias, particularly given that nearly all included studies lacked blinding. It also reflects symptom improvement rather than objective rhythm outcomes. Therefore, the observed benefit may overestimate the true treatment effect, and this outcome should be interpreted as a measure of symptomatic improvement rather than definitive antiarrhythmic efficacy. Although the definitions of overall clinical response rate varied across trials, all shared a common core of improvement in AF-related symptoms. Subgroup analysis by definition type showed consistent effects, and sensitivity analyses supported the robustness of the pooled estimate. Nevertheless, because most included studies lacked blinding, the possibility of detection bias cannot be excluded. For the overall clinical response rate, the pooled estimate was based on a mixed population, with only one postoperative study; therefore, the result mainly reflects non-surgical patients, and the effect in postoperative patients remains uncertain.

The EHRA symptom score, a validated patient-reported outcome, also showed consistent improvements [MD = −0.47, 95% CI (−0.74, −0.19), *P* = 0.0009]. The EHRA score is an ordinal scale (I–IV), but the included studies treated it as a continuous variable by assigning numerical values to each grade; the mean difference therefore represents an average reduction of 0.47 grades. The lower bound of the 95% CI (−0.74) suggests that the true reduction could be as large as approximately three-quarters of a grade, whereas the upper bound (−0.19) suggests that it could be as small as less than one-fifth of a grade. This wide interval indicates that the magnitude of benefit may vary substantially across patients, and some may experience little or no improvement. Given that the minimal clinically important difference for the EHRA score has not been firmly established, this modest reduction should be interpreted as a possible symptomatic benefit rather than a definitive clinical improvement. This finding lends support to the overall clinical response rate as a meaningful measure of symptomatic benefit. Exploratory analyses suggested that the symptomatic benefit may be dose-dependent, because the study using 3.0 g/day showed a smaller and less consistent effect than those using the recommended 6–8 g/day; however, this finding is hypothesis-generating and requires confirmation. Given that symptom control is a primary goal of AF management, particularly for patients who remain symptomatic despite rate or rhythm control therapy, these findings are clinically relevant. For patients who cannot tolerate antiarrhythmic drugs or who decline catheter ablation, YXDJ may be considered as an adjunctive option, although this requires confirmation in adequately powered trials.

### 4.2 Rhythm control and AF recurrence

AF recurrence is one of the most clinically relevant outcomes for patients undergoing rhythm control therapy, particularly after catheter ablation. In our meta-analysis, YXDJ was associated with a lower risk of 6-month AF recurrence compared with conventional therapy alone [RR = 0.43, 95% CI (0.20, 0.90), *P* = 0.03]. The point estimate corresponds to a 57% relative reduction in recurrence risk. However, the 95% CI is wide: the lower bound (0.20) suggests that the true reduction could be as large as 80%, whereas the upper bound (0.90) suggests it could be as small as 10% and is close to the null, indicating that a clinically negligible benefit cannot be excluded. This uncertainty reflects the small number of studies and events. This observed benefit may be mechanistically linked to YXDJ’s effects on atrial electrical and structural remodeling. Experimental studies have shown that YXDJ inhibits I_Na_, I_Na-L_, and I_Ca-L_ currents, suppresses abnormal electrical activity, reduces the dispersion of action potential duration and the heterogeneity of excitation conduction, restores intracellular calcium homeostasis, and ameliorates arrhythmias induced by ischemia/reperfusion or isoproterenol (Qiu et al., 2025). These ion channel-modulating properties may contribute to the stabilization of atrial electrophysiology and reduction of arrhythmogenic substrates. Additionally, by acting on targets such as SRC and PTGS2, it exerts anti-inflammatory and anti-fibrotic effects, improving myocardial structural remodeling (Xu et al., 2026a).

Of note, one study reported the sinus rhythm maintenance rate at 3, 6, and 12 months, with no significant difference between the YXDJ and control groups (Xue et al., 2020). This finding may appear to conflict with the reduction in postoperative AF recurrence observed in three other post-ablation studies. However, this apparent discrepancy is explained by the fundamentally different nature of the two endpoints. The 6-month recurrence rate is a cumulative event indicator, capturing whether a patient experienced at least one AF episode during the entire follow-up period, whereas the sinus rhythm maintenance rate is a cross-sectional snapshot typically assessed at a single time point. A reduction in cumulative recurrence indicates that YXDJ lowers the likelihood of experiencing any postoperative AF event during the vulnerable early phase, whereas the absence of a difference in cross-sectional sinus rhythm maintenance may reflect that some patients with transient recurrences were restored to sinus rhythm by the 6-month assessment. Furthermore, the sinus rhythm maintenance data were derived from a single non-surgical population (Xue et al., 2020), whereas the recurrence rate analysis was based on three post-ablation studies (Zhang et al., 2019; Zhu and Zhang, 2023; Zhu and Zhang, 2024), which makes direct comparisons across these endpoints inherently limited by population heterogeneity.

### 4.3 Cardiac structure and function

Our meta-analysis showed that YXDJ was associated with improvements in both LAEF [MD = 2.11%, 95% CI (0.70, 3.51), *P* = 0.003] and LVEF [MD = 4.39%, 95% CI (2.86, 5.92), *P* < 0.00001], suggesting a possible cardioprotective effect. For LAEF, the lower bound of the 95% CI (0.70%) suggests that the true improvement could be as small as less than 1%, which may be clinically marginal, whereas the upper bound (3.51%) suggests it could be as large as approximately 3.5%, which may be clinically meaningful. For LVEF, the lower bound (2.86%) still represents a clinically appreciable improvement, and the upper bound (5.92%) suggests a substantial benefit. The 95% CI for LVEF was relatively narrow, indicating greater precision, whereas the CI for LAEF was wider and heterogeneity was moderate to substantial (I^2^ = 71%), reflecting uncertainty about the magnitude of benefit. These improvements indicate that YXDJ may favorably influence both atrial and ventricular systolic function.

Recent integrative research combining metabolomics and network pharmacology identified that the core components of YXDJ—including liquiritigenin, liquiritin, rhamnioside D, ginsenoside Re, and ginsenoside Rg1—exert cardioprotective effects by modulating SRC and PTGS2 targets and participating in pathways such as histidine and sphingolipid metabolism (Xu et al., 2026a). Furthermore, YXDJ has been shown to inhibit the cAMP/PKA/ACTC1 signaling pathway, thereby alleviating tachyarrhythmia-induced myocardial injury and cardiac dysfunction (Xu et al., 2026b). In a myocardial ischemia-reperfusion injury model, YXDJ corrects abnormal nitrogen metabolism in myocardial tissue and reduces harmful amine substances and nitrogen free radical content, which may help restore normal energy metabolism in cardiomyocytes and alleviate myocardial tissue damage (Wang J. et al., 2021).

Regarding LAD, the overall pooled result showed no significant difference between groups [MD = −1.97 mm, 95% CI (−4.08, 0.13), *P* = 0.07], with considerable heterogeneity (I^2^ = 89%). The lower bound of the 95% CI (−4.08 mm) suggests that the true reduction could be as large as approximately 4 mm, which may be clinically meaningful, whereas the upper bound (0.13 mm) is close to the null and even crosses zero, indicating that a small increase in LAD cannot be excluded. This wide interval reflects the small number of studies and substantial heterogeneity. Sensitivity analysis suggested that the effect may be more pronounced in postoperative patients and with longer treatment duration, whereas no significant effect was observed in the non-surgical subgroup. These findings suggest that the beneficial effect of YXDJ on left atrial reverse remodeling may be more evident in patients with more advanced atrial structural abnormalities and may require longer treatment to become fully manifest. The mitral E/A ratio showed no significant improvement [MD = 0.12, 95% CI (−0.01, 0.24), *P* = 0.06]. The lower bound of the CI (−0.01) is essentially at the null, and the upper bound (0.24) suggests that a modest improvement cannot be excluded, but because the confidence interval includes zero, the evidence remains inconclusive. The heterogeneity was largely explained by a study using a lower YXDJ dosage. After excluding this study, the result remained non-significant. The limited number of studies and the wide confidence interval suggest that the current evidence is insufficient to draw a definitive conclusion regarding YXDJ’s effect on diastolic function.

### 4.4 Inflammatory biomarkers

Systemic inflammation plays a pivotal role in the pathogenesis and perpetuation of AF. Inflammatory cytokines such as hs-CRP, TNF-α, and IL-6 have been consistently associated with AF occurrence, persistence, and post-ablation recurrence (Wu et al., 2013). Our meta-analysis showed that YXDJ was associated with reductions in all three inflammatory markers compared with conventional therapy alone. For TNF-α, the SMD was used because units differed across studies; for hs-CRP and IL-6, the MD was used because units were consistent.

For hs-CRP, the mean difference was −1.21 mg/L (95% CI -1.63 to −0.78, *P* < 0.00001). The lower bound (−1.63 mg/L) suggests that the true reduction could be as large as approximately 1.6 mg/L, which would represent a clinically meaningful anti-inflammatory effect, whereas the upper bound (−0.78 mg/L) suggests it could be as small as less than 1 mg/L, which may still be clinically relevant given that hs-CRP is a sensitive marker. For TNF-α, the standardized mean difference was −0.44 (95% CI -0.71 to −0.18, P = 0.0009), representing a small-to-moderate effect size; the lower bound (−0.71) suggests a moderate-to-large effect, whereas the upper bound (−0.18) is close to a small effect. For IL-6, the mean difference was −10.42 ng/L (95% CI -14.58 to −6.26, *P* < 0.00001). The lower bound (−14.58 ng/L) suggests a substantial reduction, whereas the upper bound (−6.26 ng/L) still represents a clinically appreciable decrease. However, the confidence intervals for all three markers were relatively wide, reflecting the small number of studies and between-study heterogeneity. The reductions in hs-CRP, TNF-α, and IL-6 suggest a possible anti-inflammatory effect of YXDJ.

For TNF-α and IL-6, all included RCTs were conducted in postoperative patients, and YXDJ was associated with reductions in both markers. For hs-CRP, however, the analysis included both postoperative and non-surgical patients. Subgroup analysis showed a reduction in hs-CRP in the postoperative subgroup, whereas the reduction in the non-surgical subgroup did not reach statistical significance. Therefore, the anti-inflammatory effect of YXDJ appears to be more evident in postoperative patients, but it cannot be broadly attributed to all AF populations. These findings suggest that its mechanism in preventing early recurrence may involve inhibition of the excessive inflammatory response associated with surgery and AF itself, thereby stabilizing the atrial substrate. From a clinical perspective, the reduction in inflammatory biomarkers carries prognostic implications. Elevated hs-CRP and IL-6 levels have been shown to predict AF recurrence after catheter ablation (Boyalla et al., 2022), and IL-6 trans-signaling has been shown to contribute to AF development and is associated with AF occurrence (Li et al., 2023). Therefore, the reductions in these markers observed in our meta-analysis may not only reflect symptom improvement but also suggest a potential long-term benefit in reducing AF burden and associated cardiovascular risk. However, detection methods and sampling time points for hs-CRP and IL-6 were not uniformly reported across the included studies, which may limit the comparability of MD estimates; this should be considered when interpreting the pooled effects for these biomarkers.

Existing studies have shown that YXDJ alleviates isoproterenol-induced cardiomyocyte pyroptosis in rats with ischemic arrhythmias by inhibiting the ROS-NLRP3-GSDMD pathway, thereby protecting myocardial structural integrity (Tian et al., 2025). In addition, YXDJ reduces inflammatory cytokine levels and oxidative stress in the hearts of model rats by modulating the TAK1/MKK3/p38 MAPK signaling pathway (Li et al., 2024). Several components of YXDJ may also contribute to AF-related inflammation. In an isoproterenol-induced rat model of atrial fibrillation, glycyrrhetinic acid inhibited PIM1 expression in cardiac fibroblasts and reduced IL-4, IL-6, IL-13, and IL-17 levels, thereby attenuating atrial fibrosis (Liang et al., 2024). Ginsenoside Rg1 decreased serum TNF-α and IL-6 levels in isoproterenol-induced myocardial hypertrophy rats by inhibiting the NF-κB pathway (Lu et al., 2014). Ginsenoside Re reduced IL-6, TNF-α, and CXCL1/2/3 levels in isolated perfused rat hearts with isoproterenol-induced arrhythmia via modulation of the cAMP/PKA pathway (Cao et al., 2021). Cinnamaldehyde pretreatment reduced IL-6, IL-1β, and TNF-α levels in a rat myocardial ischemia/reperfusion injury model by inhibiting NLRP3 inflammasome activation and GSDMD-mediated pyroptosis (Luan et al., 2022). However, these mechanisms are mainly derived from animal or cell studies and have not been directly validated in patients with AF. Future studies integrating pharmacokinetics, network pharmacology, and clinical samples are needed to clarify which components of YXDJ reach effective concentrations in humans and are truly involved in IL-6 downregulation.

### 4.5 Safety

Six of the 10 included RCTs reported adverse events. The pooled analysis showed no significant difference between groups in the overall risk of adverse events [RR = 0.58, 95% CI (0.32, 1.04), *P* = 0.07]. The point estimate suggests that YXDJ may be associated with a 42% relative reduction in adverse-event risk. However, the 95% CI is wide: the lower bound (0.32) suggests that the true reduction could be as large as 68%, whereas the upper bound (1.04) crosses the null, indicating that a small increase in risk cannot be excluded. This uncertainty reflects the small number of studies and events. This finding is broadly consistent in direction with previous meta-analyses, which reported fewer adverse events in the YXDJ group (Wang XY. et al., 2021; Ji et al., 2022). However, the small number of studies, the limited sample size for safety outcomes, and the more focused AF-specific population may have reduced statistical power. Therefore, the current evidence only indicates that no clear increase in adverse-event risk was observed; it is insufficient to support definitive conclusions such as “YXDJ is safe” or that YXDJ has a comparable safety profile to conventional therapy alone.

The two studies reporting heart rate showed no significant between-group difference either at baseline or after treatment (Liu et al., 2020; Xue et al., 2020). Both groups exhibited comparable reductions in heart rate, indicating that YXDJ did not confer additional rate-limiting effects beyond conventional therapy. This neutral effect may be clinically relevant for AF patients already receiving β-blockers or those with baseline bradycardia, as YXDJ appears unlikely to exacerbate bradycardia. However, this observation is preliminary and requires confirmation in larger studies.

In addition, the exploratory data raise the possibility of a long-term cerebrovascular benefit, but the evidence is insufficient to draw definitive conclusions. The available report does not allow verification of whether anticoagulation and other stroke risk factors were adequately adjusted for. Therefore, the ischemic stroke finding should be interpreted as hypothesis-generating rather than confirmatory.

### 4.6 Comparison with previous meta-analyses

Several previous meta-analyses have evaluated the efficacy of YXDJ for arrhythmias. Wang et al. (2021) and Ji et al. (2022) reported that YXDJ combined with conventional therapy was associated with improvements in clinical response rate and LVEF, as well as reductions in heart rate and inflammatory markers in patients with various arrhythmias (Wang XY. et al., 2021; Ji et al., 2022). Yang et al. similarly reported favorable efficacy for tachyarrhythmia and potential cost-effectiveness advantages (Yang et al., 2024). However, these meta-analyses included heterogeneous arrhythmia populations, such as premature ventricular contractions, paroxysmal supraventricular tachycardia, and AF, rather than focusing specifically on AF. Because different arrhythmia types have distinct pathophysiological mechanisms and treatment responses, pooling them may obscure AF-specific effects. In contrast, our meta-analysis focused exclusively on AF, thereby improving clinical homogeneity. It also provides a more comprehensive evaluation of YXDJ in AF, including AF recurrence at 6 months, EHRA symptom scores, echocardiographic parameters (LAEF, LAD, E/A ratio), and inflammatory biomarkers (TNF-α, IL-6). However, the focused approach limits the number of eligible studies and our ability to assess publication bias, underscoring the exploratory nature of our findings.

### 4.7 Clinical heterogeneity and rationale for pooling

The included studies differed in several important clinical characteristics. First, four trials were conducted in post-ablation patients (Jiang and Lu, 2025; Zhang et al., 2019; Zhu and Zhang, 2023; Zhu and Zhang, 2024), whereas the remaining six enrolled non-surgical AF populations, including patients with heart failure, hyperthyroidism, hypertension, and non-valvular AF. Second, AF subtypes and underlying conditions varied: most studies enrolled paroxysmal AF, whereas Zhang GR (2019) (Zhang et al., 2019) included both persistent and paroxysmal AF, Liu TT (2020) (Liu et al., 2020) enrolled patients with chronic heart failure and AF with a prolonged R-R interval, and Xiao KH (2020) (Xiao and Sun, 2020) enrolled patients with hyperthyroidism-induced AF. Third, background therapy differed substantially, including amiodarone, warfarin, irbesartan, propylthiouracil plus metoprolol, sacubitril/valsartan, and other conventional treatments. Fourth, the recommended daily dose of YXDJ is 6–8 g; however, two trials used a lower dose of 3.0 g/day (Jiang and Lu, 2025; Zhu and Zhang, 2023), while the remaining eight used 6.0–8.0 g/day. Fifth, treatment duration ranged from 1 month to 12 months, with most studies using approximately 3 months.

Despite this clinical heterogeneity, we considered an overall pooled analysis to be justified for several reasons. All included studies evaluated the same research question—whether adding YXDJ to conventional therapy provides additional benefit in patients with AF—and all used the same proprietary product (YXDJ, Approval No. Z19991082). We used random-effects models when statistical heterogeneity was present, and we performed pre-specified subgroup analyses by surgical status, treatment duration, dosage, and definition type where the number of studies allowed, as well as sensitivity analyses excluding single studies that differed in key clinical characteristics. These analyses showed that the direction of effect was generally consistent across subgroups, although the small number of studies in each category limited statistical power. We acknowledge that clinical heterogeneity may still limit the interpretability of pooled estimates.

### 4.8 Chemical composition and batch consistency

Our meta-analysis suggested that YXDJ may have clinical benefits as an adjunctive therapy for AF; however, clarifying its active constituents remains important. The current national drug standard specifies the content determination of glycyrrhizic acid and uses thin-layer chromatography for the identification of ginsenoside Rg1 and cinnamaldehyde. Beyond these quality-control requirements, several analytical studies have further characterized the chemical constituents and metabolite profiles of YXDJ. For chemical profiling, a UPLC-Q-TOF-MS/MS analysis identified 51 compounds: 20 from Glycyrrhizae Radix et Rhizoma (Gancao), 11 from Ginseng Radix et Rhizoma Rubra (Hongshen), 16 from Rehmanniae Radix (Dihuang), 2 from Cinnamomi Ramulus (Guizhi), 1 from Zingiberis Rhizoma Recens (Shengjiang), and 1 shared by Glycyrrhizae Radix et Rhizoma (Gancao), Rehmanniae Radix (Dihuang), and Jujubae Fructus (Dazao) (Shi et al., 2022). Another UPLC-Q-Exactive-Orbitrap HRMS study identified 122 chemical constituents and further screened 19 active components by network pharmacology (Xu et al., 2026b). For quantitative analysis, an HPLC method was established for the simultaneous determination of liquiritin, glycyrrhizic acid, cinnamic acid, and cinnamaldehyde (Wang et al., 2017); an HPLC wavelength-switching method determined ginsenoside Rg1, ginsenoside Re, ginsenoside Rb1, cinnamic acid, and cinnamaldehyde (Pang et al., 2019); and HPLC-MS/MS quantified five core components—liquiritigenin, liquiritin, rehmannioside D, ginsenoside Re, and ginsenoside Rg1 (Xu et al., 2026a).

However, all included RCTs reported using YXDJ from the same manufacturer, but none reported the batch number, specification, chemical fingerprint, marker compound content, or purity data. Because YXDJ is composed of nine botanical and animal-derived ingredients, its chemical composition may be influenced by raw material quality, harvest season, processing methods, extraction and manufacturing conditions, and batch-to-batch variation. This potential batch-to-batch heterogeneity could affect clinical outcomes, but it could not be evaluated in the present systematic review.

### 4.9 Limitations

Several limitations should be considered. First, most included studies were rated as having some concerns or high risk of bias, mainly due to inadequate reporting of allocation concealment, lack of blinding for subjective outcomes, and absence of trial registrations or protocols; these limitations may overestimate the intervention effect. Second, the overall clinical response rate was defined using different thresholds across trials, and some definitions incorporated objective cardiac parameters while others did not. Although all definitions reflected improvement in AF-related symptoms, subgroup and sensitivity analyses supported the robustness of the pooled estimate, but the small number of studies limited statistical power, and this outcome should be interpreted as a measure of symptomatic improvement rather than a definitive efficacy endpoint. Third, the included studies showed substantial clinical heterogeneity in patient characteristics, ablation status, AF subtype, background therapy, YXDJ dosage, and treatment duration. A low I^2^ indicates statistical consistency only and should not be interpreted as evidence of clinical homogeneity, particularly when a pooled estimate was based on only two to three small studies. In addition, postoperative and non-surgical patients could not be fully separated for all outcomes because of the limited number of studies, and background therapies differed substantially across trials, which may interact differently with YXDJ and further limit clinical exchangeability. The pooled estimates therefore represent average effects across heterogeneous populations and should not be generalized to all AF patients. In addition, all studies were conducted in China, with predominantly East Asian patients, and none reported detailed racial or ethnic composition, which limits generalizability to other populations and precludes assessment of ethnicity as an effect modifier. Fourth, because fewer than 10 studies contributed to each outcome, publication bias could not be formally assessed; given that all included studies were small, Chinese-language, single-center trials, the risk of publication bias cannot be excluded. Fifth, the ischemic stroke finding was derived from a single small study with only eight events, and the multivariable model and adjusted covariates were not clearly reported; this precludes any conclusion about an independent effect beyond conventional anticoagulation. Similarly, the 9-month follow-up data from two studies, although suggesting sustained benefits, should be considered exploratory. Sixth, safety data were limited by incomplete reporting, as the included studies did not consistently specify whether adverse events were reported as patient counts or event counts, or whether the same patient experienced multiple events; furthermore, hard clinical endpoints such as cardiovascular mortality or rehospitalization were not reported, precluding assessment of long-term prognosis. Seventh, although all RCTs reported the same manufacturer and approval number of YXDJ, specifications were reported in only a minority of studies and were not consistently described, and none reported batch numbers, chemical fingerprints, marker compound content, purity, or detailed quality-control data. Because YXDJ is derived from botanical materials, its composition may vary with raw material quality, processing, and batch-to-batch variation; although the national drug standard specifies glycyrrhizic acid content and TLC identification, the impact of this heterogeneity on clinical outcomes could not be assessed. Finally, the limited number of studies precluded a formal dose-response meta-regression, leaving the optimal dosing strategy for different AF subtypes unresolved.

### 4.10 Clinical implications and future prospects

Although the current evidence is insufficient to support routine clinical use of YXDJ, these findings may inform its future positioning as an adjunctive therapy in AF management. In clinical practice, YXDJ should not replace guideline-directed anticoagulation, rate or rhythm control, or catheter ablation. However, in selected patients who remain symptomatic despite conventional therapy, or in postoperative patients with elevated inflammatory markers, YXDJ may be considered as a complementary option if future trials confirm its efficacy and safety.

For such integration to occur, several steps are needed. First, multicenter, double-blind, placebo-controlled RCTs with hard clinical endpoints, including stroke, hospitalization, cardiovascular mortality, and quality of life, are required, and these trials should also compare different doses and treatment durations. Second, future studies should report standardized chemical fingerprints, marker compound content, batch numbers, and quality-control data to ensure reproducibility, and should include pharmacokinetic, pharmacodynamic, and drug-interaction studies to clarify how YXDJ components interact with antiarrhythmic drugs and anticoagulants. Third, real-world studies and cost-effectiveness analyses will be important to evaluate its applicability in routine practice.

If these evidence gaps are addressed, YXDJ could potentially be integrated into AF management as a standardized adjunctive therapy, particularly for symptom control and postoperative inflammation, rather than as a replacement for conventional treatment.

## 5 Conclusion

The available evidence, although limited by methodological shortcomings, provides only preliminary signals that YXDJ may offer possible additional benefits as an adjunct to conventional therapy for symptom control and postoperative management in AF. The certainty of evidence ranged from very low to moderate: moderate for LVEF and 6-month postoperative AF recurrence; low for LAEF, the postoperative LAD subgroup, inflammatory biomarkers (hs-CRP, TNF-α, and IL-6), and adverse events; and very low for overall clinical response rate, EHRA symptom score, overall LAD, and mitral E/A ratio. The safety profile remains uncertain because of the limited number of RCTs and adverse events; current evidence only suggests that no clear increase in adverse-event risk was observed. These findings should be interpreted as exploratory and hypothesis-generating. YXDJ should not be used as a replacement for guideline-directed anticoagulation, rate or rhythm control, or catheter ablation. Rigorously designed, multicenter, double-blind, placebo-controlled trials are needed before any clinical recommendation can be made.

## Data Availability

The original contributions presented in the study are included in the article/Supplementary Material, further inquiries can be directed to the corresponding authors.

## References

[B1] Boyalla V. Harling L. Snell A. Kralj-Hans I. Barradas-Pires A. Haldar S. (2022). Biomarkers as predictors of recurrence of atrial fibrillation post ablation: an updated and expanded systematic review and meta-analysis. Clin. Res. Cardiol. 111 (6), 680–691. 10.1007/s00392-021-01978-w 34999932 PMC9151522

[B2] Cao Y. Y. Du B. X. Li S. H. Yuan S. Tao Y. J. Liu L. P. (2021). Effect of ginsenoside re on isoproterenol-induced cardiac arrhythmia in isolated perfused rat heart. Chin. Tradit. Herb. Drugs 52 (20), 6234–6244. 10.7501/j.issn.0253-2670.2021.20.013

[B3] ChenY. WangL. LiY. AR. OuYangJ. JiangZ. (2025). Efficacy and safety of shensong yangxin capsules for persistent atrial fibrillation: a Systematic Review and meta-analysis of randomized controlled trials. Front. Pharmacol. 16, 1620340. 10.3389/fphar.2025.1620340 40606602 PMC12213544

[B4] Guyatt G. H. Oxman A. D. Vist G. E. Kunz R. Falck-Ytter Y. Alonso-Coello P. (2008). GRADE: an emerging consensus on rating quality of evidence and strength of recommendations. BMJ 336 (7650), 924–926. 10.1136/bmj.39489.470347.AD 18436948 PMC2335261

[B5] Heinrich M. Jalil B. Abdel-Tawab M. Echeverria J. Kulić Ž. McGaw L. J. (2022). Best Practice in the chemical characterisation of extracts used in pharmacological and toxicological research-The ConPhyMP-Guidelines. Front. Pharmacol. 13, 953205. 10.3389/fphar.2022.953205 36176427 PMC9514875

[B6] Ji Z. C. Hu H. Y. Li K. Wang H. Cao L. J. Zhang J. H. (2022). Effect and safety of Yangxin Dingji Capsule combined with Western medicine for cardiac function indexes among patients with arrhythmia:a meta-analysis. Tianjin J. Tradit. Chin. Med. 39 (10), 1277–1288. 10.11656/j.issn.1672-1519.2022.10.11

[B7] Jiang X. C. Lu L. (2025). Application of Yangxin Dingji Capsule and amiodarone hydrochloride tablets in the treatment of patients with paroxysmal atrial fibrillation post-radiofrequency ablation. Chin. Sci. J. Database Citation Ed. Med. Health 7, 45–48.

[B8] Li X. Wu X. Chen X. Peng S. Chen S. Zhou G. (2023). Selective blockade of interleukin 6 trans-signaling depresses atrial fibrillation. Heart Rhythm. 20 (12), 1759–1770. 10.1016/j.hrthm.2023.08.026 37633428

[B9] Li M. Zhang Z. Li X. Y. Tian X. Zheng W. L. Wu J. W. (2024). Mechanism ofYangxin dingji capsules in preventing ventricular arrhythmia based on TAK1/MKK3/p38 MAPK pathway. Chin. J. Exp. Tradit. Med. Formulae 30 (20), 86–95. 10.13422/j.cnki.syfjx.20240438

[B10] Liang H. Zhang N. Zhao L. Wang Q. S. An Y. Wang H. (2024). Glycyrrhetinic acid alleviates atrial fibrillation by inhibiting the expression of PIM1. Chem. Biol. Drug Des. 103 (2), e14448. 10.1111/cbdd.14448

[B11] Liu T. T. Chen T. Xue J. Qi Q. Zhao L. Jiang Z. A. (2020). Clinical study on Yangxin Dingji capsule in the treatment of atrial fibrillation and chronic heart failure with long RR interval. Mod. J. Integr. Tradit. Chin. West Med. 29 (5), 468–472. 10.3969/j.issn.1008-8849.2020.05.003

[B12] Lu M. L. Wang H. X. Yang J. Zhang J. (2014). The effect of NF-κB signaling pathway on ginsenoside Rg1 inhibiting myocardial hypertrophy induced by isoproterenol in rats. Pharmacol. Clin. Chin. Mater. Med. 30 (1), 20–22. 10.13412/j.cnki.zyyl.2014.01.007

[B13] Luan F. Lei Z. Peng X. Chen L. Peng L. Liu Y. (2022). Cardioprotective effect of cinnamaldehyde pretreatment on ischemia/reperfusion injury *via* inhibiting NLRP3 inflammasome activation and gasdermin D mediated cardiomyocyte pyroptosis. Chem. Biol. Interact. 368, 110245. 10.1016/j.cbi.2022.110245 36341777

[B14] PageM. J. McKenzieJ. E. BossuytP. M. BoutronI. HoffmannT. C. MulrowC. D. (2021). The PRISMA 2020 statement: an updated guideline for reporting systematic reviews. Bmj 372, n71. 10.1136/bmj.n71 33782057 PMC8005924

[B15] Pang B. Q. Zhu Y. Q. Zhang L. (2019). Determination of five components in YangXinDingJi preparations by HPLC with wavelength switching. Chin. J. Pharm. 50 (7), 792–795. 10.16522/j.cnki.cjph.2019.07.015

[B16] Qi J. Yang Z. F. Wang W. J. Bo M. M. Wu Y. Z. Guo L. L. (2024). Meta-analysis of effect of Wenxin Granule on heart structural remodeling and inflammatory markers in patients with atrial fibrillation. Drug Eval. Re 47 (9), 2130–2146. 10.7501/j.issn.1674-6376.2024.09.022

[B17] Qiu S. Sun J. Su S. Wu W. Zhang J. Qi J. (2025). Traditional Chinese Medicine YangxinDingji alleviates arrhythmias through inhibition of sodium and L-type calcium channels. J. Ethnopharmacol. 347, 119803. 10.1016/j.jep.2025.119803 40239882

[B18] Ruddox V. Sandven I. Munkhaugen J. Skattebu J. Edvardsen T. Otterstad J. E. (2017). Atrial fibrillation and the risk for myocardial infarction, all-cause mortality and heart failure: a systematic review and meta-analysis. Eur. J. Prev. Cardiol. 24 (14), 1555–1566. 10.1177/2047487317715769 28617620 PMC5598874

[B19] Shi S. Chu Y. Jia Q. Hu Y. (2021). Comparative efficacy and safety of wenxin granule combined with antiarrhythmic drugs for atrial fibrillation: a protocol for a systematic review and network meta-analysis. Med. Baltim. 100 (3), e24434. 10.1097/md.0000000000024434 33546090 PMC7837853

[B20] Shi M. Ren B. N. Wu Y. (2022). Study on chemical constituents of Yangxin dingji capsules by UPLC-Q-TOF-MS/MS. J. Chin. Med. Mater. 45 (4), 870–876. 10.13863/j.issn1001-4454.2022.04.019

[B21] SunJ. LuY. BaoY. YinD. QuX. (2025). Global epidemiological transition of atrial Fibrillation/Flutter (1990-2021): multidimensional burden dynamics and socioeconomic health gradients across 204 countries and territories. Rev. Cardiovasc Med. 26 (12), 45091. 10.31083/rcm45091 41524042 PMC12781011

[B22] Tian X. Wang H. Yang W. Y. Liu K. Feng Y. M. Qian B. Z. (2025). Yangxin dingji capsules inhibit ISO-Induced ischemic arrhythmia by regulating ROS-NLRP3-GSDMD pathway. Pharmacol. Clin. Chin. Mater. Med. 41 (3), 41–47. 10.13412/j.cnki.zyyl.20241104.008

[B23] Wang H. X. Li L. F. An R. Cheng X. M. Wang C. H. Wang X. H. (2017). Simultaneous determination of four constituents in Yangxin Dingji Capsules by HPLC. Chin. Tradit. Pat. Med. 39 (1), 98–101. 10.3969/j.issn.1001-1528.2017.01.019

[B24] Wang X. Y. Hu H. Y. Ji Z. C. Zhai J. B. Liu C. X. Zhang J. H. (2021a). Systematic review and meta-analysis of efficacy and safety of Yangxin Dingji Capsules in treatment of arrhythmia. Zhongguo Zhong Yao Za Zhi 46 (20), 5418–5427. 10.19540/j.cnki.cjcmm.20210409.502 34738445

[B25] Wang J. Jia L. M. Liu C. Luan Y. Y. Jia M. (2021b). Protective effect of Yangxin dingji capsule on Myocardial Ischemia Reperfusion Injury. Hebei Med. 27 (7), 1191–1197. 10.3969/j.issn.1006-6233.2021.07.029

[B26] WangY. LiuY. YanX. WangD. (2022). An overview of systematic reviews on the effectiveness of Wenxin Keli in the treatment of atrial fibrillation. Evid. Based Complement. Altern. Med. 2022, 6973151. 10.1155/2022/6973151 35677364 PMC9170393

[B27] Wolfes J. Ellermann C. Frommeyer G. Eckardt L. (2025). Comparison of the latest ESC, ACC/AHA/ACCP/HRS, and CCS Guidelines on the management of atrial fibrillation. JACC Clin. Electrophysiol. 11 (4), 836–849. 10.1016/j.jacep.2024.12.018 39985521

[B28] Wu N. Xu B. Xiang Y. Wu L. Zhang Y. Ma X. (2013). Association of inflammatory factors with occurrence and recurrence of atrial fibrillation: a meta-analysis. Int. J. Cardiol. 169 (1), 62–72. 10.1016/j.ijcard.2013.08.078 24095158

[B29] Xiao K. H. Sun X. M. (2020). Effects of Yangxin dingji capsule on thyroid hormone and left atrial electrophysiological parameters in patients with hyperthyroidism-induced atrial fibrillation. Guid. J. Tradit. Chin. Med. Pharm. 26 (12), 62–4+8. 10.13862/j.cnki.cn43-1446/r.2020.12.017

[B30] Xu F. Gao F. Tian J. Mo X. Xie Z. Liu X. (2026a). Experimental the cardiac protective mechanism of YangxinDingji capsule against arrhythmia based on metabolomics and network pharmacology. J. Pharm. Biomed. Anal. 268, 117201. 10.1016/j.jpba.2025.117201 41175498

[B31] Xu F. Gao F. Tian J. Mo X. Xie Z. Liu X. (2026b). Integrating transcriptomics and network pharmacology to investigate YangxinDingji Capsule alleviates myocardial injury in tachyarrhythmia rat. J. Ethnopharmacol. 356, 120837. 10.1016/j.jep.2025.120837 41183692

[B32] Xue J. Zhao L. Qin H. Wang Y. L. Chen C. Li J. Y. (2020). Clinical Study on low-dose amiodarone combined with Yangxin dingji capsule in the treatment of elderly patients with paroxysmal atrial fibrillation. Chin. J. Integr. Med. Cardio Cerebrovasc. Dis. 18 (17), 2839–2841. 10.12102/j.issn.1672-1349.2020.17.022

[B33] Yang H. Y. Wang X. Zhang S. Q. (2024). Meta analysis and cost-effectiveness of Yangxin dingji capsule in the treatment of tachyarrhythmia. China J. Pharm. Econ. 19 (6), 19–24. 10.12010/j.issn.1673-5846.2024.06.003

[B34] Ying L. H. Liu H. Y. Zhu Y. S. (2021). Clinical Study on Yangxin Dingji Capsule combined with Irbesartan in the treatment of hypertension complicated with paroxysmal atrial fibrillation. Chin. J. Integr. Med. Cardio Cerebrovasc. Dis. 19 (13), 2222–2225. 10.12102/j.issn.1672-1349.2021.13.024

[B35] Zhu B. C. Zhang J. X. (2023). Application effect of Yangxin Dingji capsules combined with amiodarone hydrochloride tablets for patients with paroxysmal atrial fibrillation after radiofrequency ablation. Chin. J. Clin. Ration. Drug Use 16 (33), 1–4+9. 10.15887/j.cnki.13-1389/r.2023.33.001

[B36] Zhang G. R. Zhang J. Du C. Wang L. J. Jiang P. (2019). Effect of Yangxin Dingji capsule combined with amiodarone on prognosis of patients with atrial fibrillation undergoing radiofrequency ablation. Mod. J. Integr. Tradit. Chin. West Med. 28 (34), 3786–90+810. 10.3969/j.issn.1008-8849.2019.34.006

[B37] Zhang G. R. Liu T. Guo A. J. (2020). Observation on the effect of Yangxin dingji capsule in preventing ischemic stroke in patients with non-valvular atrial fibrillation. Chin. J. Integr. Med. Cardio Cerebrovasc. Dis. 18 (1), 116–118. 10.12102/j.issn.1672-1349.2020.01.029

[B38] Zhao Z. R. (2026). Effects of Yangxin Dingji capsules combined with Sacubitril/Valsartan in treatment of patients with paroxysmal atrial fibrillation. Med. J. Chin. People’s Health 38 (4), 99–101+13. 10.3969/j.issn.1672-0369.2026.04.030

[B39] Zhu B. C. Zhang J. X. (2024). Study on the prevention of atrial fibrillation recurrence after circumpulmonary venous electrical isolation by using Yangxin Dingzhe Capsule of Zhonghe medical school. Guangming J. Chin. Med. 39 (9), 1778–1781. 10.3969/j.issn.1003-8914.2024.09.028

