# Supplementary material for: Efficacy and safety of Yangxin Dingji capsule as an adjunctive therapy for atrial fibrillation: a systematic review and meta-analysis of randomized controlled trials
Source: Front Pharmacol. 2026 Oct 14;17:1952214. doi: 10.3389/fphar.2026.1952214 (PMC13713822; doi:10.3389/fphar.2026.1952214)
Supplement: Supplementary file 1 [file Supplementaryfile1.docx]

**Detailed Search Process in Eight Databases**

We searched eight databases [PubMed, Embase, Web of Science, Cochrane Library, China Biology Medicine Database (CBM), Wanfang Database, China Science and Technology Journal Database (VIP) and China National Knowledge Infrastructure (CNKI)] from database inception until April 1, 2026.

Page 2 to 3: Search strategy in PubMed.

Page 4: Search strategy in Embase.

Page 5: Search strategy in Web of Science.

Page 6: Search strategy in Cochrane Library.

Page 7: Search strategy in CNKI.

Page 8: Search strategy in Wanfang.

Page 9: Search strategy in VIP.

Page 10: Search strategy in CBM.

We screened the reference lists of relevant systematic reviews and meta-analyses, but no additional eligible studies were identified. We searched [ClinicalTrials.gov](https://clinicaltrials.gov/) and the WHO International Clinical Trials Registry Platform (ICTRP) for ongoing or unpublished trials; no ongoing eligible RCTs were identified as of April 1, 2026. Conference abstracts were excluded according to our inclusion criteria, and dissertations/theses were excluded because they were not peer-reviewed. No grey literature was included.

**Supplementary Table S1. Search strategy in PubMed.**

| Search number | Query | Results |
| --- | --- | --- |
| #5 | #1 AND #4 | 0 |
| #4 | #2 OR #3 | 85,609 |
| #3 | (Atrial Fibrillations[Title/Abstract]) OR (Fibrillation, Atrial[Title/Abstract]) OR (Fibrillations, Atrial[Title/Abstract]) OR (Auricular Fibrillation[Title/Abstract]) OR (Auricular Fibrillations[Title/Abstract]) OR (Fibrillation, Auricular[Title/Abstract]) OR (Fibrillations, Auricular[Title/Abstract]) OR (Persistent Atrial Fibrillation[Title/Abstract]) OR (Atrial Fibrillation, Persistent[Title/Abstract]) OR (Atrial Fibrillations, Persistent[Title/Abstract]) OR (Fibrillation, Persistent Atrial[Title/Abstract]) OR (Fibrillations, Persistent Atrial[Title/Abstract]) OR (Persistent Atrial Fibrillations[Title/Abstract]) OR (Familial Atrial Fibrillation[Title/Abstract]) OR (Atrial Fibrillation, Familial[Title/Abstract]) OR (Atrial Fibrillations, Familial[Title/Abstract]) OR (Familial Atrial Fibrillations[Title/Abstract]) OR (Fibrillation, Familial Atrial[Title/Abstract]) OR (Fibrillations, Familial Atrial[Title/Abstract]) OR (Paroxysmal Atrial Fibrillation[Title/Abstract]) OR (Atrial Fibrillation, Paroxysmal[Title/Abstract]) OR (Atrial Fibrillations, Paroxysmal[Title/Abstract]) OR (Fibrillation, Paroxysmal Atrial[Title/Abstract]) OR (Fibrillations, Paroxysmal Atrial[Title/Abstract]) OR (Paroxysmal Atrial Fibrillations[Title/Abstract]) | 17,861 |
| #2 | "Atrial Fibrillation"[Mesh] | 80,662 |
| #1 | (YangXinDingJi[Title/Abstract]) AND (YXDJ[Title/Abstract]) | 5 |

**Supplementary Table S2. Search strategy in Embase.**

| No. | Query | Results |
| --- | --- | --- |
| #5 | #1 AND #4 | 0 |
| #4 | #2 OR #3 | 292,626 |
| #3 | 'atrial fibrillations':ab,ti OR 'fibrillation, atrial':ab,ti OR 'fibrillations, atrial':ab,ti OR 'auricular fibrillation':ab,ti OR 'auricular fibrillations':ab,ti OR 'fibrillation, auricular':ab,ti OR 'fibrillations, auricular':ab,ti OR 'persistent atrial fibrillation':ab,ti OR 'atrial fibrillation, persistent':ab,ti OR 'atrial fibrillations, persistent':ab,ti OR 'fibrillation, persistent atrial':ab,ti OR 'fibrillations, persistent atrial':ab,ti OR 'persistent atrial fibrillations':ab,ti OR 'familial atrial fibrillation':ab,ti OR 'atrial fibrillation, familial':ab,ti OR 'atrial fibrillations, familial':ab,ti OR 'familial atrial fibrillations':ab,ti OR 'fibrillation, familial atrial':ab,ti OR 'fibrillations, familial atrial':ab,ti OR 'paroxysmal atrial fibrillation':ab,ti OR 'atrial fibrillation, paroxysmal':ab,ti OR 'atrial fibrillations, paroxysmal':ab,ti OR 'fibrillation, paroxysmal atrial':ab,ti OR 'fibrillations, paroxysmal atrial':ab,ti OR 'paroxysmal atrial fibrillations':ab,ti | 21,116 |
| #2 | 'atrial fibrillation'/exp OR 'atrial fibrillation' | 292,467 |
| #1 | 'yangxindingji':ab,ti OR 'YXDJ':ab,ti | 8 |

**Supplementary Table S3. Search strategy in Web of Science.**

|  | Search Query | Results |
| --- | --- | --- |
| #1 | (TS=(yangxindingji)) OR TS=(YXDJ) | 8 |
| #2 | ((((((((((((((((((((((((TS=(atrial fibrillations)) OR TS=(fibrillation, atrial)) OR TS=(fibrillations, atrial)) OR TS=(auricular fibrillation)) OR TS=(auricular fibrillations)) OR TS=(fibrillation, auricular)) OR TS=(fibrillations, auricular)) OR TS=(persistent atrial fibrillation)) OR TS=(atrial fibrillation, persistent)) OR TS=(atrial fibrillations, persistent)) OR TS=(fibrillation, persistent atrial)) OR TS=(fibrillations, persistent atrial)) OR TS=(persistent atrial fibrillations)) OR TS=(familial atrial fibrillation)) OR TS=(atrial fibrillation, familial)) OR TS=(atrial fibrillations, familial)) OR TS=(familial atrial fibrillations)) OR TS=(fibrillation, familial atrial)) OR TS=(fibrillations, familial atrial)) OR TS=(paroxysmal atrial fibrillation)) OR TS=(atrial fibrillation, paroxysmal)) OR TS=(atrial fibrillations, paroxysmal)) OR TS=(fibrillation, paroxysmal atrial)) OR TS=(fibrillations, paroxysmal atrial)) OR TS=(paroxysmal atrial fibrillations) | 154,719 |
| #3 | #2 AND #1 | 0 |

**Supplementary Table S4. Search strategy in Cochrane Library.**

|  | Search | Hits |
| --- | --- | --- |
| #1 | (yangxindingji) OR (YXDJ) | 2 |
| #2 | (atrial fibrillations) OR (fibrillation, atrial) OR (fibrillations, atrial) OR (auricular fibrillation) OR (auricular fibrillations) OR (fibrillation, auricular) OR (fibrillations, auricular) OR (persistent atrial fibrillation) OR (atrial fibrillation, persistent) OR (atrial fibrillations, persistent) OR (fibrillation, persistent atrial) OR (fibrillations, persistent atrial) OR (persistent atrial fibrillations) OR (familial atrial fibrillation) OR (atrial fibrillation, familial) OR (atrial fibrillations, familial) OR (familial atrial fibrillations) OR (fibrillation, familial atrial) OR (fibrillations, familial atrial) OR (paroxysmal atrial fibrillation) OR (atrial fibrillation, paroxysmal) OR (atrial fibrillations, paroxysmal) OR (fibrillation, paroxysmal atrial) OR (fibrillations, paroxysmal atrial) OR (paroxysmal atrial fibrillations) | 18,572 |
| #3 | #1 AND #2 | 0 |

**Search strategy in CNKI**

(SU='养心定悸' OR TKA='养心定悸') AND (SU=('房颤' + '心房颤动' + 'Af') OR TKA=('房颤' + '心房颤动' + 'Af'))

**Supplementary Table S3. Search strategy in WanFang**

(题名或关键词:(养心定悸) OR 摘要:(养心定悸)) and (题名或关键词:(房颤 OR 心房颤动 OR Af) OR 摘要:(房颤 OR 心房颤动 OR Af))

**Supplementary Search strategy in VIP**

(M=(养心定悸) OR R=(养心定悸)) and (M=(房颤 OR 心房颤动 OR Af) OR R=(房颤 OR 心房颤动 OR Af))

**Supplementary Search strategy in CBM**

"养心定悸"[常用字段:智能] AND ( "房颤"[常用字段:智能] OR "心房颤动"[常用字段:智能] OR "Af"[常用字段:智能])

ClinicalTrials.gov

((atrial fibrillations) OR (fibrillation, atrial) OR (fibrillations, atrial) OR (auricular fibrllation) OR (auricular fibrillations) OR (fibrillation,auricular) OR (fibrilations,auricular) OR (persistent atrialfbrillation) OR (atrialfbrillation,persisten) OR (atial fbrilations,bersistent) OR (fibrillation, persistent atrial) OR (fibrllations, persistent atrial) OR (persistent atrial fibrillations) OR (familial atrialfbrillation) OR (atrial fibrllation, familia) OR (atrial fibrilations familial) OR (famiialatial fibrilations)OR (fbrilation. familial atria)DR (fibrilations, familial atrial) OR (paroxysmal atrial fibrillation) OR (atrial fibrillation, paroxysmal) OR (atrial fbrilations,baroxysmal) OR (fbrllation, paroxysmal arial) OR (fbrllations, paroxysmal atria) OR (paroxysmal atrial fbrilations)) AND

((yangxindingii) OR (YXDJ))

International Clinical Trials Registry Platform (ICTRP)

atrial fibrillation AND Yangxin Dingji

**The final literature included**

[1]姜鑫岑,卢磊.养心定悸胶囊+盐酸胺碘酮片在阵发性心房颤动患者射频消融术后治疗中的应用[J/OL].中文科技期刊数据库（引文版）医药卫生,2025(7): 45-48.

[1]Jiang X C, Lu L. Application of Yangxin Dingji Capsule and Amiodarone Hydrochloride Tablets in the Treatment of Patients with Paroxysmal Atrial Fibrillation Post-Radiofrequency Ablation[J]. Chin Sci J Database (Citation Edition) Med Health,2025, 7: 45-48.

[2]刘婷婷,陈涛,薛剑,等.养心定悸胶囊治疗伴长RR间期的心房颤动合并慢性心力衰竭的临床研究[J].现代中西医结合杂志,2020,29(05):468-472. DOI:10.3969/j.issn.1008-8849.2020.05.003.

[2]Liu T T, Chen T, Xue J, et al. Clinical study on Yangxin Dingji capsule in the treatment of atrial fibrillation and chronic heart failure with long RR interval[J]. Mod J Integr Tradit Chin West Med,2020, 29(5): 468-472. DOI:10.3969/j.issn.1008-8849.2020.05.003.

[3]肖开虎,孙叙敏.养心定悸胶囊对甲亢源性房颤患者甲状腺素及左心房电生理指标的影响[J].中医药导报,2020,26(12):62-64+68. DOI:10.13862/j.cnki.cn43-1446/r.2020.12.017.

[3]Xiao K H, Sun X M. Effects of Yangxin Dingji Capsule on Thyroid Hormone and Left Atrial Electrophysiological Parameters in Patients with Hyperthyroidism-Induced Atrial Fibrillation[J]. 2020, 26(12): 62-64+68. DOI:10.13862/j.cnki.cn43-1446/r.2020.12.017.

[4]薛剑,赵磊,秦焕,等.小剂量胺碘酮联合养心定悸胶囊治疗老年阵发性心房颤动的临床研究[J].中西医结合心脑血管病杂志,2020,18(17):2839-2841. DOI:10.12102/j.issn.1672-1349.2020.17.022.

[4]Xue J, Zhao L, Qin H, et al. Clinical Study on Low-Dose Amiodarone Combined with Yangxin Dingji Capsule in the Treatment of Elderly Patients with Paroxysmal Atrial Fibrillation[J]. Chin J Integr Med Cardio Cerebrovasc Dis,2020, 18(17): 2839-2841. DOI:10.12102/j.issn.1672-1349.2020.17.022.

[5]应良红,刘海云,朱源生.养心定悸胶囊联合厄贝沙坦治疗高血压合并阵发性心房颤动的临床研究[J].中西医结合心脑血管病杂志,2021,19(13):2222-2225. DOI:10.12102/j.issn.1672-1349.2021.13.024.

[5]Ying L H, Liu H Y, Zhu Y S. Clinical Study on Yangxin Dingji Capsule Combined with Irbesartan in the Treatment of Hypertension Complicated with Paroxysmal Atrial Fibrillation[J]. Chin J Integr Med Cardio Cerebrovasc Dis,2021, 19(13): 2222-2225. DOI:10.12102/j.issn.1672-1349.2021.13.024.

[6]张国茹,刘涛,郭安君.养心定悸胶囊预防非瓣膜病心房颤动病人缺血性脑卒中发生的效果观察[J].中西医结合心脑血管病杂志,2020,18(01):116-118. DOI:10.12102/j.issn.1672-1349.2020.01.029.

[6]Zhang G R, Liu T, Guo A J. Observation on the Effect of Yangxin Dingji Capsule in Preventing Ischemic Stroke in Patients with Non-Valvular Atrial Fibrillation[J]. Chin J Integr Med Cardio Cerebrovasc Dis,2020, 18(1): 116-118. DOI:10.12102/j.issn.1672-1349.2020.01.029.

[7]张国瑞,张静,杜超,等.养心定悸胶囊联合胺碘酮对心房颤动射频消融术患者预后的影响[J].现代中西医结合杂志,2019,28(34):3786-3790+3810. DOI:10.3969/j.issn.1008-8849.2019.34.006

[7]Zhang G R, Zhang J, Du C, et al. Effect of Yangxin Dingji capsule combined with amiodarone on prognosis of patients with atrial fibrillation undergoing radiofrequency ablation[J]. Mod J Integr Tradit Chin West Med,2019, 28(34): 3786-3790+3810. DOI:10.3969/j.issn.1008-8849.2019.34.006

[8]朱保成,张佳秀.养心定悸胶囊联合盐酸胺碘酮片在阵发性心房颤动患者射频消融术后治疗中的应用效果[J].临床合理用药,2023,16(33):1-4+9. DOI:10.15887/j.cnki.13-1389/r.2023.33.001.

[8]Zhu B C, Zhang J X. Application effect of Yangxin Dingji capsules combined with amiodarone hydrochloride tablets for patients with paroxysmal atrial fibrillation after radiofrequency ablation[J]. Chin J Clin Rational Drug Use,2023, 16(33): 1-4+9. DOI:10.15887/j.cnki.13-1389/r.2023.33.001.

[9]朱保成,张佳秀.中和医派养心定悸胶囊预防环肺静脉电隔离术后房颤复发的研究[J].光明中医,2024,39(09):1778-1781. DOI:10.3969/j.issn.1003-8914.2024.09.028.

[9]Zhu B C, Zhang J X. Study on the Prevention of Atrial Fibrillation Recurrence after Circumpulmonary Venous Electrical Isolation by Using Yangxin Dingzhe Capsule of Zhonghe Medical School[J]. Guangming J Chin Med,2024, 39(9): 1778-1781. DOI:10.3969/j.issn.1003-8914.2024.09.028.

[10]赵自然.养心定悸胶囊联合沙库巴曲缬沙坦治疗阵发性房颤患者的效果[J].中国民康医学,2026,38(4):99-101+113. DOI:10.3969/j.issn.1672-0369.2026.04.030.

[10]Zhao Z R. Effects of Yangxin Dingji capsules combined with Sacubitril/Valsartan in treatment of patients with paroxysmal atrial fibrillation[J]. Med J Chin People’s Health, 2026,38(4):99-101+113. DOI:10.3969/j.issn.1672-0369.2026.04.030.

**Excluded literature**

Non-RCT (n = 1)

[1]韩爱,张宝田.养心定悸胶囊治疗快速性心律失常的临床研究[J].影像研究与医学应用,2017,1(12):239-240. DOI: 10.3969/j.issn.2096-3807.2017.12.151

[1]Han A, Zhang B T. Clinical study on Yangxin Dingji capsules in the treatment of tachyarrhythmia[J]. Journal of Imaging Research and Medical Applications, 2017, 1(12): 239-240. DOI: 10.3969/j.issn.2096-3807.2017.12.151

Non-YXDJ (n = 6)

[1]闫龙美.高血压合并房颤患者临床特征及调肝益气定悸方改善心房重构的研究[D].北京中医药大学,2025.DOI:10.26973/d.cnki.gbjzu.2025.000063.

[1]Yan L M. Clinical characteristics of patients with hypertension complicated with atrial fibrillation and study on Tiaogan Yiqi Dingji Formula improving atrial remodeling[D]. Beijing: Beijing University of Chinese Medicine, 2025. DOI:10.26973/d.cnki.gbjzu.2025.000063.

[2]孙翠,许祖建.养心定悸安神方治疗持续性房颤合并睡眠障碍临床观察[J].中医药临床杂志,2023,35(8):1593-1596.DOI:10.16448/j.cjtcm.2023.0830.

[2] Sun C, Xu Z J. Clinical Observation of Yangxin Dingji Anshen Formula on Persistent Atrial Fibrillation Combined with Sleep Disorders[J]. Clinical Journal of Traditional Chinese Medicine, 2023, 35(8): 1593-1596. DOI:10.16448/j.cjtcm.2023.0830.

[3]潘超群,张琪.养心定悸方治疗心房颤动的疗效观察[J].湖北中医药大学学报,2015,17(1):71-72. DOI: 10.3969/j.issn.1008-987x.2015.01.24

[3] Pan C Q, Zhang Q. Clinical Observation of Yangxindingji Decoction in Treatment of Atrial Fibrillation[J]. Journal of Hubei University of Chinese Medicine, 2015, 17(1): 71-72. DOI: 10.3969/j.issn.1008-987x.2015.01.24

[4]孟庆峰,刘春玉.养心定悸汤治疗气虚血瘀型阵发性房颤60例[J].河南中医,2014,34(10):1900-1901.DOI:10.16367/j.issn.1003-5028.2014.10.022.

[4] Meng Q F, Liu C Y. Yangxin Dingji Decoction in the treatment of 60 cases of paroxysmal atrial fibrillation of qi deficiency and blood stasis type[J]. Henan Traditional Chinese Medicine, 2014, 34(10): 1900-1901. DOI:10.16367/j.issn.1003-5028.2014.10.022.

[5]罗芳,林良智.中药配合小剂量地高辛治疗老年快速型心房纤颤疗效观察[J].吉林中医药,2007,(5):20.DOI:10.13463/j.cnki.jlzyy.2007.05.013.

[5] Luo F, Lin L Z. Observation on Therapeutic Effects of Senile Rapid Atrial Fibrillation with Chinese Drugs in Combination with Digoxin in Small Dosage[J]. Jilin Journal of Chinese Medicine, 2007, (5): 20. DOI:10.13463/j.cnki.jlzyy.2007.05.013.

[6]邢月朋,张伟,于惠卿,等.养心定悸冲剂治疗快速性心律失常的临床研究[J].中国中西医结合急救杂志,2004,(4):218-220. DOI: 10.3321/j.issn:1008-9691.2004.04.004

[6] Xing Y P, Zhang W, Yu H Q, et al. Clinical research of the curative effect of Yangxin Dingji medicinal granules(养心定悸冲剂) on rapid arrhythmia[J]. Chinese Journal of Integrated Traditional and Western Medicine in Intensive and Critical Care, 2004, (4): 218-220. DOI: 10.3321/j.issn:1008-9691.2004.04.004

Not AF-only (n = 3)

[1]杭燕雯.养心定悸胶囊治疗糖尿病性冠心病合并心律失常的疗效分析[J].中国处方药,2021,19(5):84-87. DOI: 10.3969/j.issn.1671-945X.2021.05.041

[1] Hang Y W. Clinical effect of Yangxin Dingji Capsule on diabetic coronary heart disease with arrhythmia[J]. Journal of China Prescription Drug, 2021, 19(5): 84-87. DOI: 10.3969/j.issn.1671-945X.2021.05.041

[2]李小旺,柳元化,陆建忠.养心定悸胶囊联合美西律治疗冠心病合并心律失常的疗效及对心功能的影响[J].现代实用医学,2019,31(6):737-739. DOI: 10.3969/j.issn.1671-0800.2019.06.015

[2] Li X W, Liu Y H, Lu J Z. Clinical efficacy of Yangxin Dingji Capsule combined with mexiletine in the treatment of coronary heart disease complicated with arrhythmia and its effect on cardiac function[J]. Modern Practical Medicine, 2019, 31(6): 737-739. DOI: 10.3969/j.issn.1671-0800.2019.06.015

[3]高立威.养心定悸胶囊联合美托洛尔治疗冠心病心律失常疗效观察[J].中医学报,2018,33(8):1524-1527.DOI:10.16368/j.issn.1674-8999.2018.08.361.

[3] Gao L W. Therapeutic Effect Observation of Yangxin Dingji Capsule Combined with Metoprolol in Treatment of Coronary Heart Disease Arrhythmias[J]. China Journal of Chinese Medicine, 2018, 33(8): 1524-1527. DOI:10.16368/j.issn.1674-8999.2018.08.361.
