# Supplementary material for: Efficacy and safety of Yangxin Dingji capsule as an adjunctive therapy for atrial fibrillation: a systematic review and meta-analysis of randomized controlled trials
Source: Front Pharmacol. 2026 Oct 14;17:1952214. doi: 10.3389/fphar.2026.1952214 (PMC13713822; doi:10.3389/fphar.2026.1952214)
Supplement: Supplementary file 2 [file Supplementaryfile2.docx]

**Supplementary Material S2.1**

**Botanical, zoological and pharmaceutical nomenclature of the constituent drugs of Yangxin Dingji Capsule reported in the included studies**

The scientific name, naming authority and family of every constituent drug were validated against the Medicinal Plant Names Services (MPNS, Royal Botanic Gardens, Kew) and Plants of the World Online (POWO). Drugs of animal (†) origin, which are not covered by MPNS/POWO, were verified against the Integrated Taxonomic Information System (ITIS) and mammalian taxonomy references. Pharmaceutical (drug) names and medicinal parts follow the Pharmacopoeia of the People’s Republic of China (2025 edition).

| **No.** | **Common name (Pinyin)** | **Validated scientific name (with naming authority)** | **Family** | **Pharmaceutical (drug) name** | **Medicinal part** |
| --- | --- | --- | --- | --- | --- |
| 1 | Dihuang | *Rehmannia glutinosa* (Gaertn.) Libosch. ex DC. | Orobanchaceae | *REHMANNIAE RADIX* | Fresh or dried tubers |
| 2 | Maidong | *Ophiopogon japonicus* (Thunb.) Ker Gawl. | Asparagaceae | *OPHIOPOGONIS RADIX* | Dry root tubers |
| 3 | Hongshen | *Panax ginseng* C.A.Mey. | Araliaceae | *GINSENG RADIX ET RHIZOMA RUBRA* | Dried roots and rhizomes after steaming |
| 4 | Dazao | *Ziziphus jujuba* Mill. | Rhamnaceae | *JUJUBAE FRUCTUS* | Dry ripe fruit |
| 5 | Ejiao† | *Equus asinus* Linnaeus | Equidae | *ASINI CORII COLLA* | Solid glue made by boiling and concentrating dried or fresh skin |
| 6 | Heizhima | *Sesamum indicum* L. | Pedaliaceae | *SESAMI SEMEN NIGRUM* | Dry and mature seeds |
| 7 | Guizhi | *Cinnamomum cassia* (L.) J.Presl | Lauraceae | *CINNAMOMI RAMULUS* | Dry Twig (young branch) |
| 8 | Ganjiang | *Zingiber officinale* Roscoe | Zingiberaceae | *ZINGIBERIS RHIZOMA* | Dried rhizome |
| 9 | Gancao / Zhigancao | *Glycyrrhiza uralensis* Fisch. ex DC. | Fabaceae | *GLYCYRRHIZAE RADIX ET RHIZOMA* | Dried roots and rhizomes |

**Notes.** † Drug of animal origin (zoological).

**Supplementary Material S2.2**

**The major active components and their chemical structures of YXDJ.**

| **No.** | **Component** | **Source herb** | **Category** | **Molecular formula** | **CAS No.** | **Chemical structure** |
| --- | --- | --- | --- | --- | --- | --- |
| 1 | Glycyrrhizic acid | glycyrrhizae radix et rhizoma (Gancao) | National standard, [2] | C42H62O16 | 1405-86-3 |  |
| 2 | Liquiritin | glycyrrhizae radix et rhizoma (Gancao) | [1-2] | C21H22O9 | 551-15-5 |  |
| 3 | liquiritigenin | glycyrrhizae radix et rhizoma (Gancao) | [1] | C15H12O4 | 578-86-9 |  |
| 4 | Cinnamic acid | Cinnamomi Ramulus (Guizhi) | [2-3] | C9H8O2 | 140-10-3 |  |
| 5 | Cinnamaldehyde | Cinnamomi Ramulus (Guizhi) | National standard, [2-3] | C9H8O | 104-55-2 |  |
| 6 | Ginsenoside Rg1 | ginseng radix et rhizoma rubra (Hongshen) | National standard, [3] | C42H72O14 | 22427-39-0 |  |
| 7 | Ginsenoside Re | ginseng radix et rhizoma rubra (Hongshen) | [1, 3] | C48H82O18 | 52286-59-6 |  |
| 8 | Ginsenoside Rb1 | ginseng radix et rhizoma rubra (Hongshen) | [1, 3] | C54H92O23 | 41753-43-9 |  |
| 9 | Rhmannioside D | Rehmanniae Radix (Dihuang) | [1] | C27H42O20 | 81720-08-3 |  |

In addition, the 122 constituents identified by UPLC-Q-Exactive-Orbitrap HRMS can be obtained from Supplementary Table S3 of that article [4]. The 51 constituents identified by another UPLC-Q-TOF-MS/MS analysis can be found in Table 1 of the original article [5].

References

[1].	Xu F, Gao F, Tian J, Mo X, Xie Z, Liu X, Guo Q. Experimental the cardiac protective mechanism of YangxinDingji capsule against arrhythmia based on metabolomics and network pharmacology. J Pharm Biomed Anal. 2026; 268: 117201. doi:10.1016/j.jpba.2025.117201.

[2].	Wang HX, Li LF, An R, Cheng XM, Wang CH, Wang XH. Simultaneous determination of four constituents in Yangxin Dingji Capsules by HPLC. Chin Tradit Patent Med. 2017; 39(1): 98-101. doi:10.3969/j.issn.1001-1528.2017.01.019.

[3].	Pang BQ, Zhu YQ, Zhang L. Determination of Five Components in YangXinDingJi Preparations by HPLC with Wavelength Switching. Chin J Pharm. 2019; 50(7): 792-5. doi:10.16522/j.cnki.cjph.2019.07.015.

[4].	Xu F, Gao F, Tian J, Mo X, Xie Z, Liu X, Guo Q. Integrating transcriptomics and network pharmacology to investigate YangxinDingji Capsule alleviates myocardial injury in tachyarrhythmia rat. J Ethnopharmacol. 2026; 356: 120837. doi:10.1016/j.jep.2025.120837.

[5].	Shi M, Ren BN, Wu Y. Study on Chemical Constituents of Yangxin Dingji Capsules by UPLC-Q-TOF-MS/MS. J Chin Med Mater. 2022; 45(4): 870-6. doi:10.13863/j.issn1001-4454.2022.04.019.
