# Supplementary material for: Efficacy and safety of Yangxin Dingji capsule as an adjunctive therapy for atrial fibrillation: a systematic review and meta-analysis of randomized controlled trials
Source: Front Pharmacol. 2026 Oct 14;17:1952214. doi: 10.3389/fphar.2026.1952214 (PMC13713822; doi:10.3389/fphar.2026.1952214)
Supplement: Supplementary file 3 [file Supplementaryfile3.docx]

**Supplementary figures for sensitivity analysis and subgroup analysis of the outcome**

**S1 Overall clinical response rate**

S1.1 Sensitivity analysis using random-effects model

S1.2 Sensitivity analysis excluding Zhu BC 2023

S1.3 Sensitivity analysis excluding Liu TT 2020

S1.4 Sensitivity analysis excluding Xiao KH 2020

S1.5 Sensitivity analysis excluding Ying LH 2021

S1.6 Sensitivity analysis excluding Zhao ZR 2026

**S2 EHRA symptom score**

S2.1 Sensitivity analysis excluding Zhu BC 2023 (fixed-effect)

S2.2 Sensitivity analysis excluding Zhu BC 2023 (random-effect)

**S3 Postoperative recurrence of AF at 6 months**

S3.1 Sensitivity analysis using random-effects model

S3.2 Sensitivity analysis excluding Zhu BC 2023

**S4 LAEF**

S4.1 Sensitivity analysis excluding Zhu BC 2024

S4.2 Sensitivity analysis excluding Xue J 2020

S4.3 Sensitivity analysis excluding Zhu BC 2023

**S5 LVEF**

S5.2 Sensitivity analysis using random-effects model

S5.2 Sensitivity analysis excluding Jiang XC 2025

S5.3 Sensitivity analysis excluding Liu TT 2020

**S6 LAD**

S6.1 Sensitivity analysis excluding Xue J 2020

S6.2 Sensitivity analysis excluding Zhu BC 2023

S6.3 Sensitivity analysis excluding Liu TT 2020

**S7 Mitral E/A ratio**

S7.1 Sensitivity analysis excluding Zhu BC 2023

**S8 hs-CRP**

S8.1 Sensitivity analysis excluding Jiang XC 2025

S8.2 Sensitivity analysis excluding Zhao ZR 2026

S8.3 Sensitivity analysis excluding Xue J 2020

**S9 TNF-α**

S9.1 Sensitivity analysis using random-effects model

S9.2 Sensitivity analysis excluding Zhang GR 2019 (fixed-effect)

**S10 IL-6**

S10.1 Sensitivity analysis excluding Jiang XC 2025

S10.2 Sensitivity analysis excluding Zhang GR 2019

**S11 Adverse events**

S11.1 Sensitivity analysis of main analysis using random-effects model

S11.2 Sensitivity analysis excluding Liu TT 2020 (fixed-effect)

S11.3 Sensitivity analysis excluding Xue J 2020 and Zhang GR 2020 (fixed-effect)
